# Exposure to heavy metal stress triggers changes in plasmodesmatal permeability via deposition and breakdown of callose

**DOI:** 10.1093/jxb/ery171

**Published:** 2018-06-13

**Authors:** Ruthsabel O’Lexy, Koji Kasai, Natalie Clark, Toru Fujiwara, Rosangela Sozzani, Kimberly L Gallagher

**Affiliations:** 1 Department of Biology, University of Pennsylvania, Philadelphia, PA, USA; 2 Department of Agriculture and Life Sciences, University of Tokyo, Tokyo, Japan; 3 Department of Plant and Microbial Biology, North Carolina State University, Raleigh, NC, USA; 4 Biomathematics Graduate Program, North Carolina State University, Raleigh, NC, USA

**Keywords:** Callose, copper, iron, nutrient stress, plasmodesmata, root development

## Abstract

Both plants and animals must contend with changes in their environment. The ability to respond appropriately to these changes often underlies the ability of the individual to survive. In plants, an early response to environmental stress is an alteration in plasmodesmatal permeability with accompanying changes in cell to cell signaling. However, the ways in which plasmodesmata are modified, the molecular players involved in this regulation, and the biological significance of these responses are not well understood. Here, we examine the effects of nutrient scarcity and excess on plasmodesmata-mediated transport in the *Arabidopsis thaliana* root and identify two CALLOSE SYNTHASES and two β-1,3-GLUCANASES as key regulators of these processes. Our results suggest that modification of plasmodesmata-mediated signaling underlies the ability of the plant to maintain root growth and properly partition nutrients when grown under conditions of excess nutrients.

## Teaser

As sessile organisms, plants continually modify their growth to adapt to changes in their environment. Here we show that significant changes in plasmodesmatal permeability underlie root responses to nutrient stress.

## Introduction

Throughout their development, plants are subject to changes in their environment. Plant growth, metabolism, and development are affected by both biotic (biological) and abiotic (environmental) factors. When these factors negatively impact the health, fitness, or survival of the plant, they are referred to as biotic or abiotic stress. An increasingly common form of abiotic stress is poor soil quality, where a scarcity of essential nutrients or contamination with heavy metals decreases plant productivity and generally decreases plant health (Bates, 1971; Nagajyoti *et al.*, 2010). Plants have developed several strategies to mitigate the negative impacts of poor soil on growth. Among these strategies are the ability to adjust (increase or decrease) nutrient uptake (Takahashi *et al.*, 1997; Grotz *et al.*, 1998; Vert *et al.*, 2001; Sancenón *et al.*, 2004), modify growth to increase or limit access to nutrients (Caldwell and Pearcy, 1994; Williamson *et al.*, 2001; Linkohr *et al.*, 2002; Lequeux *et al.*, 2010; Gruber *et al.*, 2013), and the ability to mobilize and sequester excess nutrients to prevent toxicity and cell death (Salt *et al.*, 1995; Seregin *et al.*, 2003).

A common acute response of plants to biotic or abiotic stress is a decrease in plasmodesmatal permeability (Sivaguru *et al.*, 2000; Samardakiewicz *et al.*, 2012). Nearly all cells in the plant are connected to their neighbors by plasmodesmata, intercellular channels that physically connect the plasma membrane and endoplasmic reticulum (ER) of the adjoining cells, creating cytoplasmic (symplastic) continuity (Robards and Lucas, 1990). Throughout the life of the plant, plasmodesmata serve as major conduits for the movement of sugars and metabolites to fuel growth, as well as the trafficking of developmental signals that control tissue patterning and cell identity (Kurata *et al.*, 2005; Corbesier *et al.*, 2007; Koizumi *et al.*, 2012; Kawade, 2014). Many small molecules are able to diffuse freely through plasmodesmata; however, the extent of symplastic movement of a molecule depends both upon its size and the size of the plasmodesmatal aperture (or plasmodesmatal pore) (Wolf *et al.*, 1989; Angell *et al.*, 1996; Kim *et al.*, 2002; Roberts and Oparka, 2003; Zambryski, 2004). Plasmodesmatal permeability therefore is often measured as the size of a molecule that can move freely between neighboring cells and the distance that the molecule is able to travel from its source. During the course of normal development in *Arabidopsis thaliana*, transient decreases in plasmodesmatal permeability are associated with the initiation of new organs and the transition from vegetative to floral growth (Gisel *et al.*, 2002; Benitez-Alfonso *et al.*, 2013). Defects in symplastic signaling are often lethal. The ability of both endogenous and environmental cues to modify plasmodesmatal permeability therefore may allow abiotic signals directly to alter developmental programs.

A major regulator of plasmodesmatal permeability is the polysaccharide (β-1,3-glucan) callose. Callose is an essential component of the phragmoplast and is present in specialized and newly formed cell walls. In mature cell walls, callose is found in association with plasmodesmata where is regulates plasmodesmatal permeability. Increases in plasmodesmata-localized callose are associated with decreases in plasmodesmatal aperture and a reduction in the free movement of molecules between cells (Zavaliev *et al.*, 2011; Vatén *et al.*, 2011). Callose levels are regulated by the opposing activities of two enzymes: callose synthases (β-1,3-glucan synthases) and β-1,3-glucanases, which produce and break down callose, respectively (Chen and Kim, 2009). While callose is an important player in developmental and environmental responses, very little is known about how the callose synthases and β-1,3-glucanases are regulated. Increased levels of intracellular calcium and reactive oxygen species have been linked to both increases and decreases in callose and changes in plasmodesmatal permeability (Holdaway-Clarke *et al.*, 2000; Benitez-Alfonso *et al.*, 2011; Steinhorst and Kudla, 2013). Additionally, there appears to be subfunctionalization of the callose synthases both in where they function within the cell and in their responses to biotic and abiotic stress (Jacobs *et al.*, 2003; Cui and Lee, 2016).

Recently Müller *et al.* (2015) showed that growth of *A. thaliana* seedlings on medium lacking phosphate caused a significant increase in the accumulation of callose in the root meristem, particularly surrounding the quiescent center (QC) cells. Accompanying the increase was a reduction in plasmodesmatal permeability and alterations in cellular patterning. Because the phosphate starvation phenotype could be rescued by reducing the levels of iron in the medium, it was suggested that the increase in callose induced by low phosphate is an iron toxicity response. These results raise the possibility that increased levels of essential nutrients translate into changes in development via alterations in symplastic signaling.

In order to better understand the connections between abiotic stress, callose, plasmodesmatal permeability, root growth, and nutrient distribution, we examined the acute effects of nutrient stress (paucity and excess) on cell to cell signaling in the root of *A. thaliana*. In examining the effects of various growth conditions on callose deposition and root growth, we found that a decrease in plasmodesmal permeability is not a universal response to an abiotic stress. Specifically, excess iron and copper caused distinct responses in *A. thaliana.* Growth of roots on excess iron inhibited the growth of the primary root and decreased plasmodesmatal permeability, whereas excess copper generally increased movement through plasmodesamata. Iron and copper exerted their effects on plasmodesmata via the regulation of specific callose synthases and β-1,3-glucanases, respectively.

## Materials and methods

### Plant materials and growth conditions

Col-0 seedlings were used as controls for all lines, with the exception of *cals6* where WS was used (Supplementary Table S1 at *JXB* online). The Cycb1;1:GUS (β-glucuronidase) lines were provided by Dr Scott Poethig (University of Pennsylvania), the SUC2:GFP (green fluorescent protein) lines were provided by Dr Steffen Abel (Leibniz Institute of Plant Biochemistry), and the *cals* lines indicated in Supplementary Table S1 were provided by Dr Jung-Youn Lee (Delaware Biotechnology Institute). All seeds were sterilized using 20% bleach solution, and kept at 4 °C for 2 d before sowing onto 1× Murashige and Skoog (MS) medium (Caisson Labs, Catalog number MSP01, Smithfield, UT) with 1% sucrose and 1% Difco Agar (BD, Catalog number 21144530, Franklin Lakes, NJ, USA). Plants were grown in an incubation chamber using a 16 h light/8 h dark cycle at 23 °C. After 5 d, seedlings were transferred to new medium for 1 d or 3 d before analysis. For plates containing excess heavy metals, the following concentrations were added to 1× MS medium: 50 µM CuSO_4_, 150 µM ZnSO_4_, 600 µM FeEDTA, or 85 µM CdCl_2_. For low nutrient plates, the medium was made with the following concentrations of macro- and micronutrients (in place of MS): 5 mM potassium nitrate, 2 mM calcium nitrate, 2 mM magnesium sulfate, 2.5 mM potassium phosphate, 14 µM manganese chloride, 70 µM boric acid, 1 µM zinc sulfate, 0.5 µM copper sulfate, 0.2 µM sodium molybdate, 0.01 µM cobalt chloride, and 50 µM NaFe EDTA. For low phosphate medium, potassium phosphate was omitted, 2.5 mM potassium chloride was added as a potassium source, and agar was purified (washed) as described in Ward *et al.* (2008). For low iron medium, NaFe EDTA was omitted, and 100 µM Ferrozine (an iron chelator) was added. For low zinc medium, ZnSO_4_ was omitted.

### Growth measurements

Five-day-old seedlings were transferred to the indicated media for 1–3 d. Growth of the primary root was measured on unmodified 1× images of roots before and after treatment using the segmented line function tool in ImageJ; two-tailed *t*-test, *P*<0.05.

### Aniline blue staining

Seedlings were grown for 5 d on standard MS medium and then transferred for 24 h to new medium as indicated. After 24 h on the new medium, roots were stained with aniline blue and assessed for the induction of callose. Seedlings were stained in aniline blue solution (750 µl of 67 mM K_3_PO_4_ pH 9.5, 240 µl of distilled water, 1 µl of Silwet-77, and 10 µl of 1% aniline blue) in the dark for 1 h prior to imaging as described in Cui and Lee (2016). Quantification of callose levels was performed on unmodified confocal micrographs of aniline blue-stained roots taken using a Leica SP5 scanning confocal microscope with a 405 laser. When quantified, the entire root was selected as a region of interest (ROI) and the mean fluorescence intensity was measured using ImageJ. For additional information on quantification of callose using aniline blue staining, see Zavaliev *et al.* (2011, 2013). For each set of experiments, control images were taken on the same day as the experimental (stress) treatments and the same confocal and confocal settings were used each time the roots were imaged. The minimum number of roots used for each experiment is given in the relevant figure legends.

### CFDA

CFDA [5(6)-carboxyfluorescein diacetate (Invitrogen, Catalog number C195, Carlsbad, CA, USA] was dissolved in DMSO at 50 mM stock concentration and stored at –20 °C. Fresh working solution was prepared for each experiment by diluting the stock 1:50 in autoclaved water. Prior to imaging, 2 µl of the CFDA working solution was applied to root tips under a dissecting scope using a 2 µl micropipette. Roots were then incubated at room temperature for 5 min and imaged on an Olympus MVX10 stereomicroscope using the GFP filter. The extent of carboxyfluorescein (CF) movement from the root tip towards the shoot was measured using thje ImageJ segmented line function. The color threshold was adjusted to minimize autoflourescence. The parameters were as follows: hue 57–108, saturation 133–255, brightness 0–60, thresholding method default, threshold color white, color space HSP (the thresholding protocol is based upon DANS methods as described in Cui *et al.*, 2015). Wilcoxon rank sum test was used; asterisks indicate *P*<0.05. The boxplot was created using R Studio.

### GUS staining

Whole seedlings were placed in ice-cold 90% acetone for 10 min. Acetone was then removed and replaced with staining buffer containing 50 mM PO_4_ buffer, 0.2% Triton-X100, and 2 mM ferrocyanide/ferricyanide, and incubated for 10 min. Staining buffer was then removed and replaced with staining buffer containing 2 mM X-Gluc and incubated for 15 min. Samples were incubated at 37 °C overnight. Samples were washed three times with 70% ethanol, and imaged using differential interference contrast (DIC).

### SUC2:GFP

Five-day-old seedlings expressing SUC2:GFP were transferred to stress medium for 1 d. Roots were then imaged using a Leica SP5 confocal microscope with a 488 laser and standard Leica defined conditions for quantifying GFP fluorescence. Propidium iodide was used as a counterstain for the cell wall. Ratiometric data were generated by taking QC:P fluorescence intensity ratios in ImageJ.

### Raster image correlation spectroscopy

Five-day-old 35S::GFP seedlings were transferred to stress medium for 24 h. Raster image correlation spectroscopy (RICS) was performed according to Clark *et al.* (2016). Images were collected using a Zeiss 710 confocal microscope. Frames were acquired using Raster scan with a dwell time of 12.61 µs pixel^–1^, for 100 frame series. Diffusion coefficients were derived using SimFCS software. Wilcoxon rank sum test was used, *P*<0.05. The boxplot was created using R Studio.

### Laser ablation-inductively coupled plasma-mass spectrometry

LA-ICP-MS was performed according to Shimotohno, *et al.* (2015). Five-day-old seedlings were treated with 50 μM Cu for 24 h, then mounted on microscope slides using double-sided tape. Positions for laser ablation were set using computer software and a camera, with a spot size of 10 µm and a spacing of 30 µm between punches. Accumulative signal intensities (accumulative for all punches in single spot, for a single root) were calculated, and then three biological replicates were used to calculate an average for each spot.

## Results

### Restrictions in plasmodesmatal permeability are not a universal response to nutrient stress

**Fig. 1.**
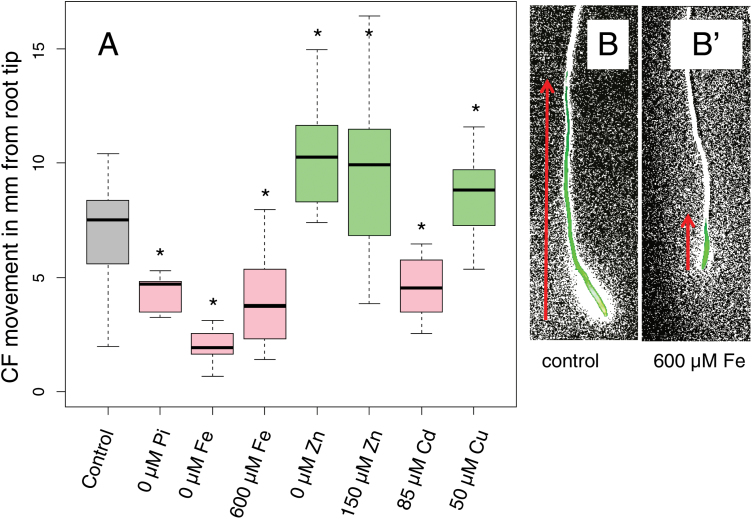
Changes in movement of CF after nutrient stress. (A) Distance of CF transport (from the root tip shootwards) in roots grown for 24 h under the treatment conditions indicated in the figure. For this assay, CFDA was micropipetted to the root tip and then incubated at room temperature for 5 min prior to imaging. Distance of movement was then measured as described in the Materials and methods on modified images using ImageJ. Green boxes indicate an increase in movement; pink indicates a decrease. A significant difference relative to control images is also indicated by asterisks (Wilcoxon rank sum test; *P*<0.05, *n*>24). (B, B'). Representative images of CF after a color threshold has been applied using ImageJ under (B) control or (B') 600 µM Fe. The red arrows indicate the direction and approximate extent of CF movement.

To determine whether a decrease in plasmodesmatal permeability is a general response to abiotic stress, we exposed 5-day-old *A. thaliana* seedlings to media lacking phosphate, iron, or zinc, as well as media containing excess iron, copper, or zinc for 24 h. In addition, we examined the effects of cadmium, which competes with these metals for its uptake into the root. The concentrations used here for excess heavy metals reflect conditions encountered by terrestrial plants grown in polluted soils (Anderson, 1991; Holmgren *et al.*, 1993; Cole *et al.*, 2001; Li *et al.*, 2001; Arias *et al.*, 2004; Kumar Sharma *et al.*, 2007). To measure plasmodesmatal permeability, we used the symplastic tracer, CFDA (Wang and Fisher, 1994; Cui *et al.*, 2015). Within plant cells, CFDA is converted into the fluorescent molecule CF, which is membrane impermeable and traffics between cells exclusively via plasmodesmata (Cui *et al.*, 2015). Under our assay conditions, when CFDA was applied to the root tip, CF was rapidly detected in the epidermis and cortex in the root meristem and within 5 min of application moved an average distance of 6.8 mm, shootwards (Fig. 1A, B). In roots grown for 24 h on 0 µM phosphate, 0 µM iron, 600 µM iron, or with 85 µM cadmium, the shootward movement of CF was significantly reduced, suggesting that these growth conditions elicited a decrease in plasmodesmatal permeability (Fig. 1A). In contrast, roots grown 0 µM zinc, 150 µM zinc, or 50 µM copper all increased the distance that CF traveled from the root tip, indicating an increase in plasmodesmatal permeability (Fig. 1A). Under all growth conditions (paucity or excess), cells in the root meristem were viable and actively dividing (*cycb1;1:GUS* expression; Supplementary Fig. S1). Likewise, with the exception of phosphate deficiency which significantly increased the frequency of emerged root hairs (Fig. 3G), none of these treatments affected the overall patterning of the root. These results suggest that the changes in the movement of CF in response to nutrient stress are not the result of general changes in the structure of the root, but rather modifications in plasmodesmatal permeability.

**Fig. 2.**
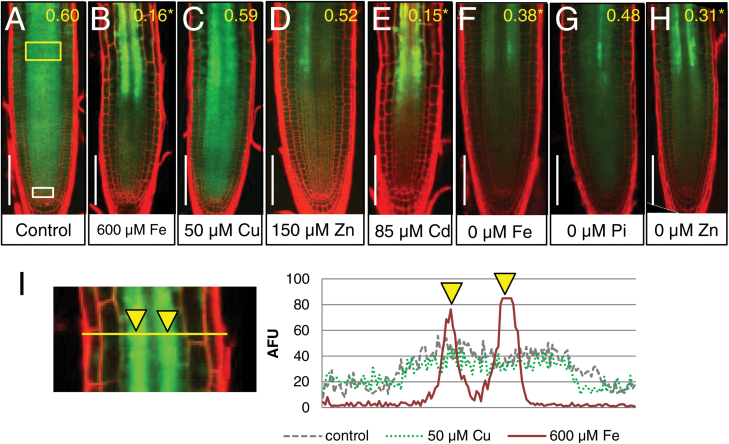
Effect of nutrient stress on the movement of GFP from the phloem. (A–H) SUC2:GFP-expressing 6-day-old seedlings 24 h after transfer to (A) standard MS (control), (B) 600 µM iron, (C) 50 µM copper, (D) 150 µM zinc, (E) 85 µM cadmium, (F) 0 µM iron, (G) 0 µM phosphate, and (H) 0 µM zinc-containing media. Scale bar=75 µm. Inset values are the ratio of mean GFP mean fluorescence intensity in the stele of the root tip just above the QC (white boxed region) relative to the stele in the transition zone of the root where SUC2 is expressed (yellow boxed region); an asterisk indicates statistical significance (*P*<0.05) in a two-tailed *t*-test, *n*>10. (I) GFP signal intensity (reported as arbitrary fluorescence units; AFU) measured radially (as indicated by the yellow line) across the phloem strands (cells where SUC2 is expressed, yellow arrows) in the transition zone of the root. The graph shows one representative sample profile each from: control (gray), 50 µM copper- (green), and 600 µM iron- (red) treated roots. Additional images are provided in Supplementary Fig. S2.

To complement the CFDA assays, we examined movement of free (untagged) GFP (~27 kDa) in the root meristem. Under normal growth conditions, GFP is able to move via plasmodesmata (Schönknecht *et al.*, 2008). When GFP is expressed in the phloem companion cells using the SUCROSE-H^+^ SYMPORTER 2 (SUC2) promoter (*SUC2:GFP*), GFP is unloaded into the pericycle and diffuses via plasmodesmata throughout the entire root meristem (Fig. 2A; Supplementary Fig. S2) (Imlau *et al.*, 1999; Ross-Elliott *et al.*, 2017). In roots with defective trafficking, however, GFP movement into the meristem is limited (Benitez-Alfonso *et al.*, 2009; Müller *et al.*, 2015). Hence the extent of movement of GFP (driven by the SUC2 promoter) from the phloem into the root meristem is commonly used as a measure of plasmodesmatal permeability (Müller *et al.*, 2015). In our assays, growth of roots for 24 h on 50 µM copper, 150 µM zinc, or 0 µM phosphate had no significant effect on movement of GFP (Fig. 2C, D, G, I; Supplementary Fig. S2B). In contrast, 24 h treatment of roots with 0 µM iron, 0 µM zinc, 600 µM iron, or 85 µM cadmium reduced GFP movement (Fig. 2B, E, F, H, I; Supplementary Fig. S2A). To quantify movement of GFP from the phloem into the meristem, we calculated the ratio of GFP fluorescence in an ROI above the QC (Fig. 2A, white box; this is a region into which GFP moves) relative to an ROI in the stele (Fig. 2A, yellow box) where GFP is directly expressed (the QC:P ratio). Additionally, we measured GFP intensity levels in the radial axis across a region of the root where the phloem strands extend into the meristem. In roots with a decreased QC:P ratio, we observed peaks in GFP signal intensity in the two phloem strands (Fig. 2I; Supplementary Fig. S2), suggesting a decrease in movement of GFP in the radial axis. These results indicate that short-term exposure of roots to iron and zinc deficiency, excess iron, or cadmium decreases plasmodematal permeability in the post-phloem domain of the root meristem and limits movement in the radial axis. Collectively these data indicate that while many stressors trigger a decrease in plasmodesmatal permeability, a restriction in plasmodesmata-mediated trafficking is not a universal response to abiotic stress.

### Changes in the accumulation of callose drive the response of the root to iron and copper

**Fig. 3.**
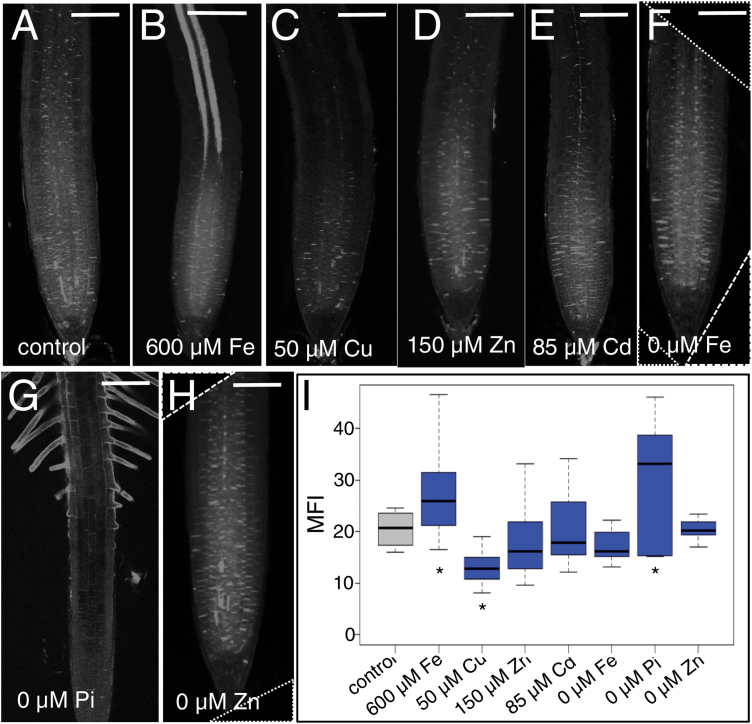
Iron, copper, and phosphate stress alter the levels of callose. Aniline blue staining of callose 24 h after transfer to (A) standard (control) MS medium, (B) 600 µM iron, (C) 50 µM copper, (D) 150 µM zinc, (E) 85 µM cadmium, (F) 0 µM iron, (G) 0 µM phosphate, and (H) 0 µM zinc. Scale bar=100 µm. (I) Mean fluorescence intensity (MFI) of the aniline blue signal was calculated in ImageJ (an unstained root is shown in Supplementary Fig. S3D to demonstrate the lack of autofluorescence). An asterisk under boxes indicates a significant difference (asterisks indicate *P*<0.05; Wilcoxon rank sum test, *n*>19) in fluorescent intensity relative to the control. In (F) and (H) images were squared off after cropping using black fill with a dotted white outline to show modification to the original image.

When callose is deposited at plasmodesmata, there is a general decrease in the size of plasmodesmatal apertures and restricted movement of macromolecules between cells. To determine if any of our growth conditions affect the accumulation of callose in the root, we stained roots with aniline blue, a fluorescent dye that quantitatively binds callose (Zavaliev and Epel, 2015). In the root meristem, callose is particularly apparent in the walls of newly divided cells; however, low basal levels of callose are found surrounding all cells under normal growth conditions (Fig. 3A). Short-term (24 h) growth of roots on 85 µM cadmium, 0 µM iron, 0 µM zinc, and 150 µM zinc had no effects on callose levels or the gross pattern of callose deposition in the root meristem, elongation, or differentiation zones when compared with controls (Fig. 3D–F, H, I). In contrast, roots grown on media with 50 µM copper had a significant decrease in the levels of callose (Fig. 3C, I), which correlated with the observed increase in movement of CF (Fig. 1). Growth of roots on phosphate-deficient media resulted in a tissue-specific accumulation of callose in the root epidermis, particularly in regions where the root hairs were present (Fig. 3G). Growth of roots on 600 µM iron resulted in a largely phloem-specific increase in the accumulation of callose (Fig. 3B). Interestingly, the deposition of callose in response to excess iron was reversible: when roots were transferred onto normal media and allowed to recover for 24 h, the distribution and levels of callose returned to normal pre-treatment levels and profiles (Supplementary Fig. S3A'–C'). The restriction of GFP movement was similarly reversible. When roots grown for 24 h on excess iron were transferred back onto control media for 24 h, the QC:P ratios returned to normal and the radial GFP profiles were identical to the pre-treatment control roots (Supplementary Fig. S3A–C). These results indicate that the block to movement of free GFP in response to excess iron is not the result of permanent changes to plasmodesmatal structure and instead is correlated with changes in callose.

**Fig. 4.**
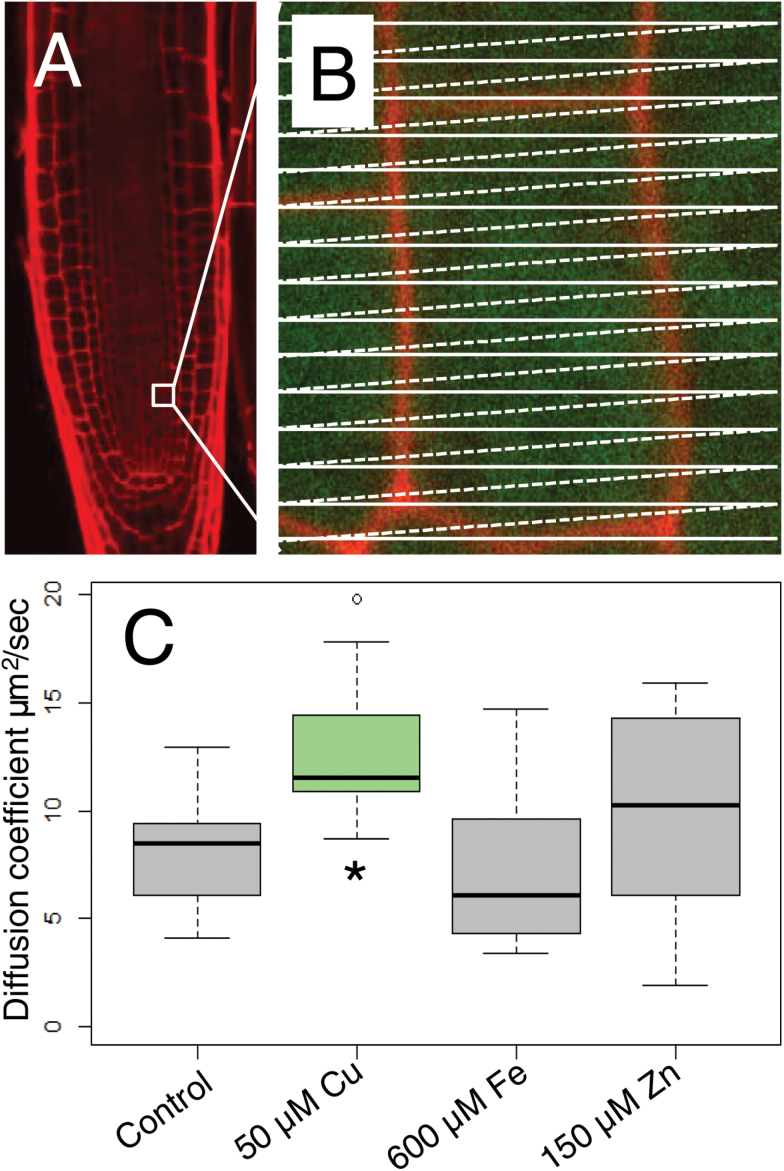
Treatment of roots with 50 µM copper increases the coefficient of diffusion of GFP in the root meristem. (A) The small white box in the propidium iodide (PI)-stained root shows a representative region of interest (ROI) used for RICS. This region is shown in (B) as a magnified image. For these assays, 5-day-old roots expressing the *35S:GFP* construct were transferred for 24 h to media containing excess copper, iron, or zinc prior to imaging. (C) Average diffusion coefficients for free GFP in roots after 24 h of treatment with 50 µM copper, 600 µM iron, or 150 µM zinc are shown in the box plots. An asterisk indicates a significant (*P*<0.05, Wilcoxon rank sum test, *n*>14) difference in movement from control roots. The circle outside the box is an outlier.

To assess further the relationship between callose and plasmodesmatal permeability, we used RICS to measure the diffusion coefficient of GFP between cells in the stele of the root meristem (Rossow *et al.*, 2010; Clark *et al.*, 2016). Specifically, we used GFP driven by a 35S promoter (*35S:GFP*) and measured the diffusion of GFP in roots grown on 50 µM copper, 600 µM iron, and 150 µM zinc. While treatment of roots with 600 µM iron restricted movement of GFP (*SUC2:GFP*) out of the phloem, it had no effect on the diffusion coefficient of GFP (7.33 μm^2^ s^–1^ in iron-treated roots relative to 7.94 μm^2^ s^–1^ in the controls; Fig. 4C), suggesting that the decrease in the QC:P ratio of the iron-treated roots (Fig. 2B) reflects an inability of GFP to move out of the phloem and not a general decrease in plasmodesmatal permeability throughout the meristem. These results are consistent with the largely tissue-specific increase in callose we see in the phloem of roots exposed to excess iron (Fig. 3B). Treatment of roots with 50 µM copper or 150 µM zinc increased the movement of CF (Fig. 1); however, only excess copper affected the diffusion coefficient of GFP within the meristem (12.74 μm^2^ s^–1^ relative to 7.94 μm^2^ s^–1^ in the controls; Fig. 4A). These data suggest that the decrease in callose deposition in response to excess copper (Fig. 4C, I) results in a general increase in plasmodesmatal permeability. In contrast, treatment of roots with 150 µM zinc (Fig. 4D, I) had no effect on the overall levels of callose in the root and limited effects on plasmodesmatal permeability.

### Callose synthases and β-1,3-glucanases control the levels of callose in response to excess iron and copper

**Fig. 5.**
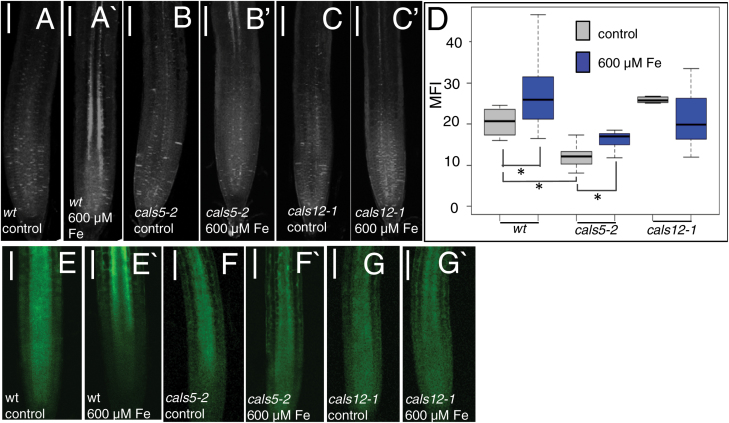
*cals5* and *cals12* seedlings have altered responses to excess iron. (A–D) Aniline blue staining of callose in (A and A') the wild type, (B and B') *cals5-2*, and (C and C') *cals12-1* under (A–C) control conditions, and (A'–C') 24 h after treatment with 600 µM Fe. (D) Quantification of callose levels in *cals5-2* and *cals12-1* roots after 24 h of growth on control medium or medium containing 600 μM Fe. Asterisks indicate *P*<0.05, two-tailed *t*-test, *n*>19. (E–G') Treatment of *cals5* and *cals12* seedlings with 600 µM Fe has no effect on the movement of GFP. GFP localization in 6-day-old wild-type, *cals5*, and *cals12* seedlings expressing SUC2:GFP 24 h after transfer to (E–G) standard MS (control) medium or (E'–G') medium containing 600 µM iron. QC:P ratios (as shown in Fig. 2) for (F( and (F') are 0.49 and 0.51, respectively, and 0.63 and 0.64 for (G) and (G'), respectively. Scale bars in A–G'=100 µm.

To identify genes involved in the response of *A. thaliana* roots to excess iron or copper, we tested the ability of callose synthase and β-1,3-glucanase mutants to respond to 24 h treatment with excess iron or copper (Supplementary Fig. S4). We were able to isolate viable homozygous loss-of-function lines for 10 of the 12 callose synthase (*CalS*) family members; loss of *CalS9* and *CalS10* are gametophytic and seedling lethal, respectively (Enns *et al.*, 2005; Huang *et al.*, 2009; Thiele *et al.*, 2009). Eight of the 10 *cals* lines responded to excess iron in the same fashion as wild-type plants, with strong and specific increases in aniline staining in the phloem (Supplementary Fig. S4). In contrast, *cals5* (*cals5-2*, *cals5-3*, or *cals5*-5) and *cals12* (*cals12-1* or *cals12-2*) showed an attenuated response to excess iron (Fig. 5). Basal levels of callose were decreased in *cals5* roots; however, the *cals5* lines were responsive to treatment with excess iron, showing an average 31% increase in aniline blue staining overall (comparable with the 33% increase in the wild type; Fig. 5D). Likewise, 24 h treatment of the *cals5* lines with excess iron significantly inhibited movement of CF (Supplementary Fig. S5). However, there were qualitative difference in how the *cals5* lines responded to excess iron. Only 50% of the *cals5* roots showed prominent phloem-specific accumulation of callose in response to excess iron. In addition, when *cals5* roots were grown for 24 h on 600 µM iron, callose levels increased, but only to the levels of untreated wild-type roots (Fig. 5D). None of the *cals5-2* lines expressing the *SUC2:GFP* marker showed any changes in the QC:P ratio of GFP in response to treatment with excess iron (Fig. 5F, F'; wild-type control images are shown in Fig. 5E, E'), which is consistent with the low basal levels of callose in *cals5* roots. The *cals12* roots showed normal basal levels of callose, but no increases in callose levels in response to 24 h treatment with excess iron (Fig. 5D). Consistent with these results, GFP movement from the phloem of the *SUC2:GFP*-expressing *cals12* roots was not inhibited by treatment with excess iron (Fig. 5G, G'). CF movement, however, was inhibited in *cals12* roots in response to excess iron (Supplementary Fig. S5). These results suggest that *CalS5* is required for basal production of callose in the root, whereas *CalS12* is responsible for iron-induced production of callose. Pathways independent of either *CalS5* or *CalS12* restrict movement of CF.

**Fig. 6.**
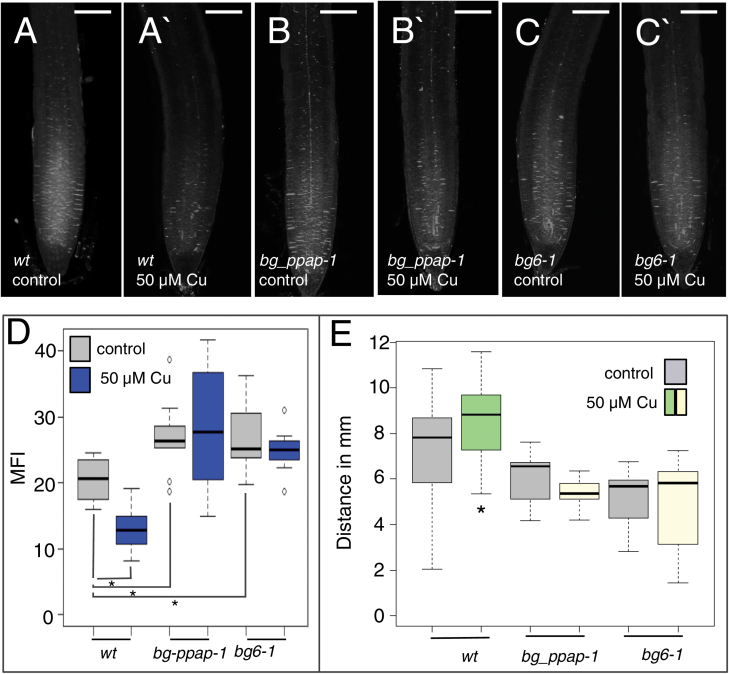
Mutations in the β-1,3-glucanases, *BG_PPAP* or *BG6*, impair the roots’ response to copper. Aniline blue staining of roots from the wild type and β-1,3-glucanase mutants (genotypes indicated) (A–C) prior to and (A'–C') 24 h after transfer to control MS medium or medium containing 50 µM Cu. Scale bar=100 µm. (D) Quantification of callose levels in the wild type, *bg_ppap-1*, and *bg6-1* before and 24 h after 50 μM Cu treatment. Asterisks indicate a significant difference (*P*<0.05, comparisons indicated; two-tailed *t*-test, *n*>19). (E) CFDA transport in *bg_ppap-1* and *bg6-1* roots is not affected by excess copper; asterisks indicate a significant difference (*P*<0.05; Wilcoxon rank sum test, *n*>24).

Next, we examined D-class (as defined by Doxey *et al.*, 2007) β-1,3-glucanases, which show root-specific expression. We were able to isolate homozygous mutants for all of the group D genes with the exception of At3g04010. In addition, we examined loss-of-function lines for BG_PPAP, a stress-responsive plasmodesmata-localized β-1,3-glucanase (Levy *et al.*, 2007) (Supplementary Table S1). Neither *bg_ppap* (*bg_ppap-1* or *bg_ppap-2*) nor *bg6* (*bg6-1* or *bg6-2*; At4g16260) showed a reduction in callose levels or an increase in CF movement in response to excess copper (Fig. 6), indicating that both BG_PPAP and BG6 are required for wild-type responses to excess copper. However, BG_PPAP and BG6 are not generally required for the breakdown of callose. Both *bg_papp-1* and *bg6-1* showed wild-type increases in phloem-specific callose in response to excess iron and, more importantly, normal breakdown of callose after 24 h recovery on normal media (Supplementary Fig. S6). These results indicate that BG_PPAP and BG6 mediate a reduction in callose levels in response to excess copper. They further support the premise that changes in callose underlie changes in plasmodesmatal permeability.

### Changes in callose levels under excess copper or iron influence root growth

**Fig. 7.**
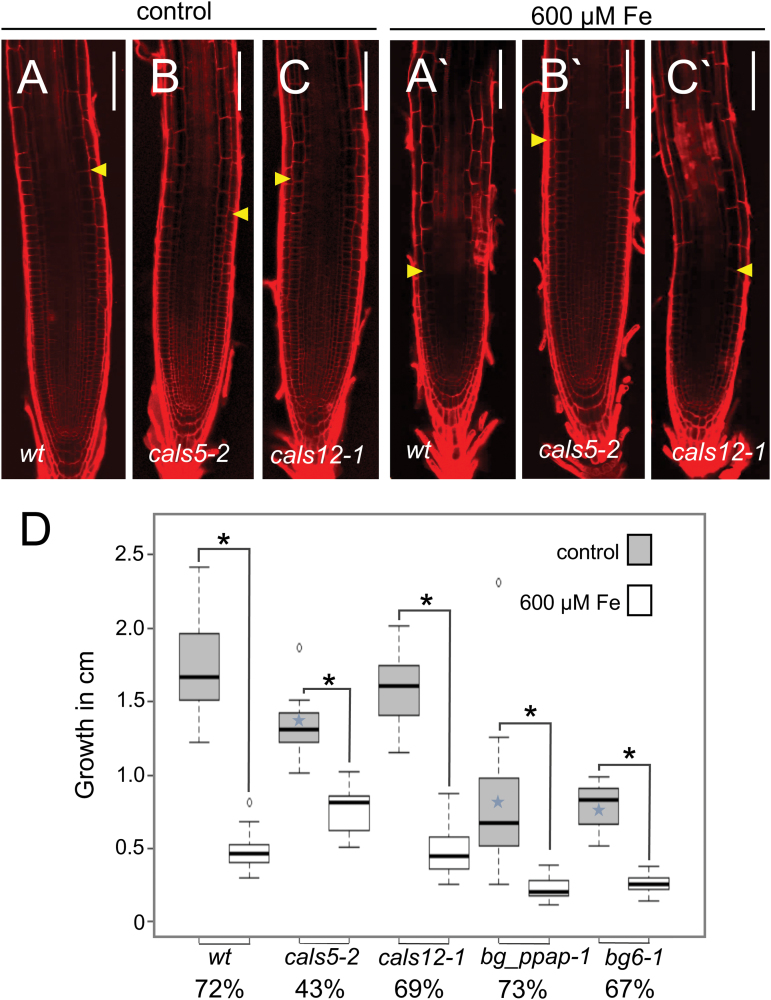
*cals5* mutants are less sensitive to the growth-inhibiting effect of 600 µM iron than the wild type. (A–C) Five-day-old roots (genotypes indicated) were transferred for 3 d to (A–C) regular MS medium or (A'–C') medium containing 600 µM iron and then stained with propidium iodide (PI) to visualize the cell walls. Scale bar=100 µm. The yellow arrowheads indicate the end of the meristematic zone and the beginning of the elongation zone. (D) Mean increase in the length of the primary root during the 3 d period post-transfer to control medium or medium containing of 600 µM iron. Percentages below the root genotypes indicate the percentage inhibition of growth relative to control roots transferred to MS medium. Two-tailed *t*-test; asterisks indicate *P*<0.05, *n*>14; error bars indicate the SE. Blue stars indicate that growth is statistically different from the wild type under control conditions.

**Fig. 8.**
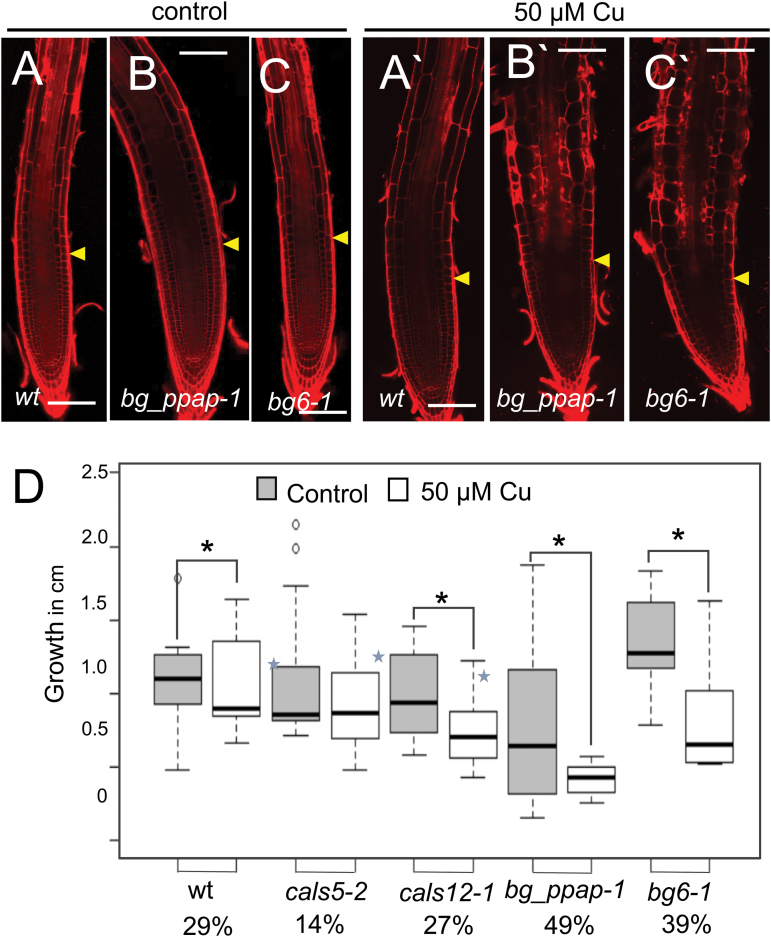
β-1,3-Glucanase mutants are more sensitive to the growth-inhibiting effect of 50 μM copper than the wild-type. (A–C) Five-day-old roots (genotypes indicated) were transferred for 3 d to (A–C) regular MS medium or (A'–C') medium containing 50 μM Cu and then stained with propidium iodide to visualize the cell walls. Scale bar = 100 μm. (D) Increase in the length of the primary root during the 3 d post-transfer to control or medium containing of 50 μM copper. Percentages below the root genotypes indicate the percentage inhibition of growth relative to MS-grown roots. Two-tailed *t*-test; asterisks indicate *P* < 0.05, *n* > 14; error bars indicate the SE. Blue stars on bars indicate that growth is statistically different (*P* < 0.05; two-tailed *t*-test) from the wild type under control conditions.

To examine the connection between nutrient stress, callose, and root growth, we looked at changes in the growth of *A. thaliana* roots after treatments with excess copper or iron. As shown in Supplementary Fig. S7, both 50 µM copper and 600 µM iron decreased the rate of root growth (by 35% and 50%, respectively, at day 1, and by 33% and 70%, respectively, at day 3); however, even after 7 d of growth, the root meristems are actively dividing (Supplementary Fig. S1A–D, inset). Several groups have shown a correlation between callose and root growth, where stress-induced increases in callose inhibit growth of the primary root (Sivaguru *et al.*, 2000; Müller *et al.*, 2015; Zhang *et al.*, 2015). To test whether reducing callose levels could ameliorate the effects of excess iron or copper on primary root growth, we measured the growth of *cals5-2* and *cals12-1* roots after 3 d on 600 µM Fe or 50 µM Cu. As shown in Figs 7 and 8, *cals5* but not *cals12* seedlings were less sensitive to iron-induced inhibition of root growth. Since the *cals5-2* lines, but not *cals12-1*, had decreased basal levels of callose (Fig. 5D), these results suggest that reducing basal levels of callose may moderate the effects of copper or iron stress on root growth.

To examine further the relationship between callose and root growth, we measured the effects of excess copper on the growth of *bg_ppap-1* and *bg6-1* roots, which fail to break down callose in response to excess copper. Relative to the wild type, the growth of *bg_ppap-1* and *bg6-1* roots was significantly inhibited by 3 d incubation on 50 µM Cu (Fig. 8D. As shown in Fig. 8A'–C'), there is a decrease in the size of the meristem of *bg_ppap-1* and *bg6-1* grown for 3 d on 50 µM Cu and evidence of cell death (for comparison *bg_ppap-1* and *bg6-1* roots 24 h after 50 µM copper treatment are shown in Supplementary Fig. S8). Cells in the elongation zone of the *bg_ppap-1* and *bg6-1* roots exposed to 50 µM Cu showed a radially swollen phenotype, indicating defects in cell expansion. Note that the *bg_ppap-1* and *bg6-1* roots were not more sensitive to the inhibitory effects of excess iron treatment (Fig. 7), indicating that these roots are not just inherently more sensitive to heavy metal stress. Collectively, these results suggest that increases in callose in response to heavy metal stress generally limit primary root growth. Moreover, the breakdown of callose by *BG_PPAP* and *BG6* helps to maintain cellular integrity and normal root growth in the presence of excess copper.

### The distribution of copper is altered in the β-1,3-glucanase mutants

**Fig. 9.**
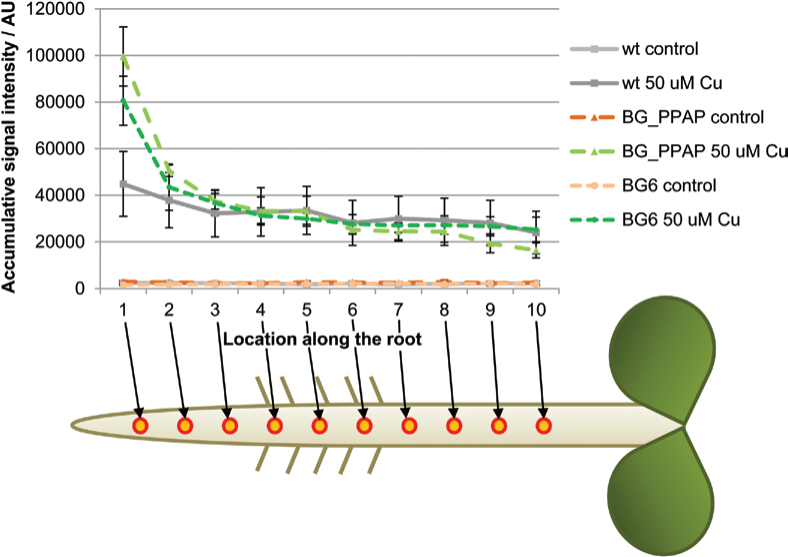
β-1,3-Glucanase mutants accumulate excess copper in the root tip. Accumulative signal intensity of copper in arbitrary units (AU) in the wild type, *bg_ppap*, and *bg6* after 24 h of 50 µM Cu; *n*=3 for each group. The *x*-axis corresponds to the location of the sample taken along the root, with punch 1 being at the meristem and moving shootwards.

Since there are both apoplastic and symplastic routes for the transport of heavy metals, changes in symplastic permeability may alter the distribution of metals within the root. To determine where in the root iron and copper accumulates and whether changes in plasmodesmatal permeability affect elemental distributions, we used LA-ICP-MS (Shimotohno *et al.*, 2015) to sample roots. For our assays, 10 μm punches (positioned along a linear grid; Fig. 9) were taken from a live *A. thaliana* root; these punches were coupled to MS to determine elemental concentrations in these tissues. Unfortunately, the counts per second for iron analysis were very low in all genotypes, in all regions of the root, and not resolvable from the background. Thus, we were unable to determine accumulative signal peak intensity for iron levels in the root. However, we were able to assay copper concentrations.

Under normal growth conditions, the wild type, *bg_ppap-1*, and *bg6-1* all showed similar levels of accumulation of copper throughout the root. After 24 h on 50 µM copper, wild-type roots showed increases in copper levels throughout the length of the root, with even higher levels in punches 1 and 2, which correspond to the meristem and elongation zones of the root. In root punches 3–10, *bg_ppap-1* and *bg6-1* roots did not differ from the wild type in the levels of copper. In punch 2, levels of copper in *bg_ppap-1* and *bg6-1* were slightly higher than in the wild type. However, in the meristem (punch 1), copper levels were dramatically (2- to 2.5-fold) higher in *ppap-1* and *bg6-1* relative to wild-type roots treated with excess copper. These results indicate that copper levels are altered in the meristem of *bg_ppap-1* and *bg6-1* roots relative to the wild type. The region of the root with the most substantial increases in copper in the *bg_ppap-1* and *bg6-1* roots at 24 h is also the region of the root whose development appears most affected by excess copper at day 3. These results suggest that in the absence of BG_PPAP or BG6, roots are less able to restrict copper from the root meristem, perhaps due to reduced symplastic movement of copper in these lines.

## Discussion

A central problem for any organism is the need to co-ordinate complex developmental and physiological programs while simultaneously responding and adapting growth to a changing environment. This is particularly true for plants, whose growth can be dramatically affected by the environment. A common response of plants to abiotic stress (wounding, cold treatment, and metal toxicity) is a restriction in plasmodesmatal permeability. Here we show that while many different types of nutrient stress (paucity or excess) affect symplastic signaling, not all conditions decreased movement through plasmodesmata. For example, treatment of *A. thaliana* seedlings with excess copper increased movement of both CF out of the root tip (Fig. 1) and free GFP within the root meristem (Fig. 4). In contrast, treatment of seedlings with 600 µM iron, 85 µM cadmium, or 0 µM iron followed the expected trend; movement of both CF and free GFP was reduced compared with controls, indicating a decrease in plasmodesmatal permeability (Figs 1, 2). For both the 50 µM copper treatment and the 600 µM iron treatment, the changes in symplastic signaling correlated with decreases and increases in the levels of callose, respectively. However, neither 0 µM iron nor 85 µM cadmium had any quantitative effects on aniline blue staining in the root meristem or elongation zone (Fig. 3). Particularly intriguing were the effects of 0 µM zinc on plasmodesmatal permeability. Growth of roots on 0 µM zinc had no effects on callose (at least as measured by aniline blue staining), but increased the movement of CF out of the root tip (Fig. 1) and decreased movement of free GFP out of the phloem into the root meristem (Fig. 2). These results suggest differential regulation of plasmodesmatal permeability (perhaps shootward versus rootward transport) in response to limiting zinc in a callose-independent manner. While callose is the best understood regulator of plasmodesmatal permeability, other molecules, subcellular domains, and structural differences can regulate cell–cell connectivity. Changes in plasmodesmata structure, microdomains, or membrane contact sites (MCSs) all influence plasmodesmatal permeability. We would not be able to detect any of these alterations using our aniline blue staining protocol.

Previously, Müller *et al.* (2015) showed that growth of *A. thaliana* seedlings for 48 h on 0 µM phosphate blocked movement of free GFP out of the phloem and decreased movement of SHR–GFP into the QC cells in the root meristem. These changes in trafficking correlated with increased callose deposition in the root meristem, particularly in the QC. While we observed a decrease in the movement of CF in response to 0 µM phosphate (Fig. 1) and increased callose deposition in the root meristem and elongation zone (Fig. 3; particularly in the root epidermis), we were not able to replicate the changes in QC-specific accumulation of callose or plasmodesmatal permeability (Müller *et al.*, 2015).

One of the suggestions of Müller *et al.* (2015) was that the response of the root (callose deposition, reduced growth, and depletion of the stem cell population) to low phosphate was an iron toxicity response. This idea is based upon the findings of Ward *et al.* (2008) who showed that the effects of low phosphate on the root meristem are ameliorated by removing iron from the media. However, neither group looked specifically at the effects of excess iron on root growth. Here, when roots were grown on media containing excess iron (100–600 µM) with sufficient phosphate, we saw a general stunting of root growth and dramatic increases in the accumulation of phloem-specific callose (Supplementary Fig. S8), but no changes in the organization or viability of the meristem. Even when germinated and grown for 7 d on media containing 600 µM iron, the cells within the meristem were mitotically active (Supplementary Fig. S1). Recently, Gutiérrez-Alanís *et al.* (2017) showed that growth of *A. thaliana* seedlings on 0 µM phosphate causes a redistribution of iron out of the QC that elicits the ectopic expression of CLAVATA3/ESR (CLE)-related protein 14 (CLE14); CLE14 in turn triggers the differentiation of the meristem independent of callose distribution. Likewise, Gutiérrez-Alanís *et al.* (2017) showed that when *A. thaliana* seedlings were grown for 7 d on medium with sufficient phosphate, but excess iron (50–500 µM), there was little effect on the root meristem and no changes in the expression of CLE14. These results indicate that excess iron and 0 µM phosphate have distinct effects on root growth.

Callose is a key regulator of plasmodesmatal permeability whose levels are controlled by the antagonistic activities of callose synthases and β-1,3-glucanases. Of the 12 annotated callose synthases in *A. thaliana*, CalS10 and CalS7 (at least in part) localize to plasmodesmata and non-redundantly regulate cell to cell trafficking (Töller *et al.*, 2008; Xie *et al.*, 2011). CalS3 also localizes to plasmodesmata, and in gain-of-function assays was shown to reduce plasmodesmatal permeability (Vatén *et al.*, 2011). Recently Cui and Lee (2016) showed that *CalS1* and *CalS8* control plasmodesmatal permeability in response to biotic (infection with *Pseudomonas syringae* pv. *maculicola*) and abiotic (wounding) stress, respectively, indicating that at least 5 of the 12 callose synthases in *A. thaliana* play a role in plasmodesmatal permeability. Here we find roles for both *CalS5* and *CalS12* in the regulation of callose and plasmodesmatal permeability. In our assays, *CalS12* specifically impaired the ability of the root to respond to iron; *cals12* mutants had diminished ability to deposit phloem-specific callose and to block movement of GFP out of the phloem in response to excess iron (Fig. 5). Unlike *CalS12*, *CalS5* was required for the basal level of callose in unstressed roots, and *cals5* mutants showed normal increases in callose in response to iron (Fig. 5), indicating that *CalS5* is not specifically required for iron-induced changes in plasmodesmatal permeability.

Three different β-1,3-glucanases (pdBG1, pdBG2, and BG_PPAP) have been shown to localize to plasmodesmata and regulate plasmodesmatal permeability (Levy *et al.*, 2007; Benitez-Alfonso *et al.*, 2013). Here we tested the role of three functionally uncharacterized β-1,3-glucanases, whose expression is highly enriched in roots, and BG_PPAP, which is the only one of the three known plasmodesmata-localized β-1,3-glucanases that is stress responsive (pdBG1 and pdBG2 play essential roles in lateral root development). *BG6* and *BG_PPAP* were non-redundantly required for copper-induced decreases in callose levels and associated increases in CF movement. Under control conditions, both the *bg_ppap* and *bg6* mutants showed elevated levels of callose, suggesting that both play some role in regulating basal callose levels in the absence of a stress response (Fig. 6). BG_PPAP, pdBG1, and pdBG2 are all glycosylphosphatidylinositol (GPI)-anchored proteins, which constitutively localize to plasmodesmata (Gaudioso-Pedraza and Benitez-Alfonso, 2014). In contrast, BG6 (At4g16260) shares similarity to the predicted pathogenesis-related (PR) β-1,3-glucanase, NtGLA. In *Nicotiana tabacum*, NtGLA is an intracellular protein that localizes to the vacuole (Zavaliev *et al.*, 2013). Despite this localization, NtGLA functions in the constitutive regulation of callose at plasmodesmata, similar to the function of BG_ PPAP in *A. thaliana*. The localization of BG6 is not known; however, it shares >50% identity with NtGLA. In the absence of a GPI anchor, Zavaliev *et al.* (2013) propose that NtGLA transiently associates with plasmodesmata by an alternative secretory pathway; similar models are possible for BG6. Despite unknown plasmodesmatal localization, BG6 both maintains basal callose levels and is required for copper-induced decreases in callose and increases in plasmodesmatal permeability in the root of *A. thaliana.*

A common response of plants to abiotic stress is an increase in callose and a stunting of root growth. Here we show that conditions that decrease callose levels in response to copper or iron stress ameliorate the inhibition of primary root growth. *cals5* lines, which have reduced basal levels of callose, are less sensitive to the inhibitory effects of both excess iron (Fig. 7) and copper (Fig. 8) on primary root growth, indicating that factors that decrease callose levels in response to heavy metal stress may buffer the negative effects on primary root growth. Consistent with this model, Zhang *et al.* (2015) recently identified a β-1,3-glucanase, SbGlu1, which is responsible for resistance to aluminum in particular cultivars of soybean. When SbGlu1 was heterologously expressed in *A. thaliana*, the SbGlu1-expressing plants accumulated less callose in response to excess aluminum and had increased resistance to aluminum as assayed by primary root growth. These results suggest that stress-induced callose inhibits root growth and that mechanisms that moderate callose levels may lessen the impact of heavy metals on primary root growth.

It should be noted that the short-term responses that we measured here might not reflect longer term effects. Under suboptimal nutrient conditions, root system architecture generally changes to increase the formation, emergence, and growth of lateral roots at the expense of the primary root. The increase in lateral branching increases the foraging capacity of the root, and in the cases of nutrient excess may allow the root to grow into less toxic soils. Classic experiments using barley grown in soils with heterogeneously distributed nutrients show that the root system dramatically increased lateral root growth and the emergence of root hairs into regions of favorable nutrient content (Drew, 1975). Thus, an inhibition of primary root growth (via the generation of reactive oxygen species, increases in the deposition of callose, or both) may represent an initial step in the release of root apical dominance that allows for an increase in lateral branching.

A surprising finding of our analysis was that in response to treatment with 50 µM copper there is a significant decrease in the levels of callose in the meristem and elongation zone of *A. thaliana* roots (Fig. 3). Previous analysis of the effects of copper on callose levels in *Silene paradoxa* indicated a correlation between the ability to break down callose in response to elevated levels of copper and copper resistance (Colzi *et al.*, 2015). When sensitive *S. paradoxa* seedlings were exposed to excess copper there was an increase (~50%) in the accumulation of callose. In contrast, in resistant plants, exposure to excess copper triggered a decrease (75%) in the callose levels. In the sensitive plants, excess copper induced root swelling and cell death, similar to what we observed in the *bg_ppap-1* and *bg6-1* roots (Fig. 8), whereas there were no morphological changes in the resistant lines. These results suggest that the ability to break down callose may be a naturally evolved mechanism to mitigate the toxic effects of elevated levels of copper.

Exposure of roots to osmotic stress is one of the few growth conditions that, similarly to excess copper, increases plasmodesmatal permeability. When grown under conditions of hyperosmolarity, there is a rapid and transient increase in plasmodesmatal permeability in *P. sativum* roots, which may allow the movement of sugars (and other osmolytes) into the root meristem and elongation zone and thus maintain normal turgor pressure and prevent plasmolysis (Schulz, 1995). In contrast, the trend in the *bg_ppap-1* and *bg6-1* mutants, which no longer show increased plasmodesmatal permeability in response to copper, is root swelling. Examination of the effects of excess copper on the cellular morphology of *Allium cepa* or *Allium sativum* (where root cells are large and easily imaged) showed a loss of the normal transverse organization of cortical microtubules in interphase cells (Liu *et al.*, 1994; Qin *et al.*, 2015). Similarly, Horiunova *et al.* (2016) reported random orientation of cortical microtubules in the epidermis of *A. thaliana* (var. Landsberg) roots after treatment with excess copper. Interestingly, of all the metals which Horiunova *et al.* (2016) tested (including cadmium, zinc, and nickel), copper most strongly disrupted the orientation of microtubules in *A. thaliana*. This is consistent with the radially swollen phenotype that we see in the *bg_ppap-1* and *bg6-1* roots after growth on excess copper and suggests that the microtubule cytoskeleton may be a particular target for copper toxicity.

Since elevated levels of copper are particularly toxic to cells, plants have evolved mechanisms to deliver copper to the appropriate subcellular compartment and thereby maintain free copper at low levels within the cytoplasm of the cell. An interesting aspect of copper homeostasis is the remobilization and redistribution of copper during leaf senescence. During the process of leaf senescence, copper ions are scavenged from the dying leaf and shunted towards regions of growth (e.g. reproductive tissues). Interestingly in *A. thaliana* there is some evidence that the copper chaperone, CCH, which functions in the mobilization of copper in senescing tissues is phloem mobile and traffics through plasmodesamata (Mira *et al.*, 2001). Therefore, conditions that increase plasmodesmatal permeability may increase the mobility of copper.

The increase in plasmodesmatal permeability in *A. thaliana* roots in response to 50 µM copper appears beneficial to the plant; when this response is attenuated, plants have poor root growth, decreased viability of the meristem, and altered distributions of copper. Given that heavy metals move symplastically at several points in their transport, decreased copper mobility (a consequence of decreased plasmodesmata permeability) could explain the 2-fold increase in copper concentrations in the root apices of the β-1,3-glucanase mutants compared with the wild type. The *bg_ppap-1* and *bg6-1* roots may be less able to move copper out of the root tip into the xylem for transport into the shoot. However, when copper accumulation in shoot tissues was examined in *A. thaliana* seedlings grown hydroponically in excess copper, there was little translocation into the shoot, indicating that sequestration of copper in shoot tissue may not be a major mechanism for detoxification of copper in *A. thaliana* (Lequeux *et al.*, 2010).

Another possibility is that *bg_ppap-1* and *bg6-1* take up more copper than wild-type roots. The uptake of copper from the soil relies significantly upon the high-affinity Cu transporter, COPT1. COPT1 is a plasma membrane-localized protein located particularly at the root tips in *A. thaliana* (Sancenón *et al.*, 2004), which is the region of the roots in which we see excess copper in the *bg_ppap-1* and *bg6-1*mutants. It is unclear how changes in symplastic connectively would regulate the acquisition of copper in the root meristem; however, both iron and phosphate homeostasis in the root meristem are regulated by the long-distance trafficking of shoot-derived proteins and RNAs (Vert *et al.*, 2003; Lin and Chiou, 2008; Pant *et al.*, 2008; Liu *et al.*, 2016). It is conceivable that similar feed-back mechanisms regulate copper.

## Supplementary data

Supplementary data are available at *JXB* online.

Table S1. Germplasm used in this study

Table S2. Effects of heavy metal stress on wild-type and mutant roots.

Fig. S1. Nutrient stress does not inhibit cell divisions.

Fig. S2. Fluorescent profile of GFP in nutrient-stressed roots.

Fig. S3. The restriction of GFP movement in iron-stressed roots is reversible.

Fig. S4. Quantification of callose in the *calS* and *β-1,3-glucanase* lines.

Fig. S5. Movement of CF is not rescued in the *cals5-2* and *cals12-1* lines.

Fig. S6. *BG_PPAP* and *BG6* are not required for recovery of roots from stress.

Fig. S7. Growth of the primary root is inhibited by excess copper or iron.

Fig. S8. β-1,3-Glucanase mutants have increased sensitivity to copper.

Supplementary Tables Figures

## References

[CIT0001] Anderson MA 1991 Long-term effects of copper rich swine manure application on continuous corn production. Communications in Soil Science and Plant Analysis 22, 993–1002.

[CIT0002] Angell SM, Davies C, Baulcombe DC 1996 Cell-to-cell movement of potato virus X is associated with a change in the size-exclusion limit of plasmodesmata in trichome cells of *Nicotiana clevelandii*. Virology 216, 197–201. 8614986 10.1006/viro.1996.0046

[CIT0003] Arias M, López E, Fernández D, Soto B 2004 Copper distribution and dynamics in acid vineyard soils treated with copper-based fungicides. Soil Science 169, 796–805.

[CIT0004] Bates TE 1971 Factors affecting critical nutrient concentrations in plants and their evaluation: a review. Soil Science 112, 116–130.

[CIT0005] Benitez-Alfonso Y, Cilia M, San Roman A, Thomas C, Maule A, Hearn S, Jackson D 2009 Control of Arabidopsis meristem development by thioredoxin-dependent regulation of intercellular transport. Proceedings of the National Academy of Sciences, USA 106, 3615–3620. 10.1073/pnas.0808717106 PMC2651306 19218459

[CIT0006] Benitez-Alfonso Y, Faulkner C, Pendle A, Miyashima S, Helariutta Y, Maule A 2013 Symplastic intercellular connectivity regulates lateral root patterning. Developmental Cell 26, 136–147. 23850190 10.1016/j.devcel.2013.06.010

[CIT0007] Benitez-Alfonso Y, Jackson D, Maule A 2011 Redox regulation of intercellular transport. Protoplasma 248, 131–140. 21107619 10.1007/s00709-010-0243-4

[CIT0008] Caldwell MM , Pearcy RW **(eds)** 1994 Exploitation of environmental heterogeneity by plants: ecophysiological processes above- and belowground. San Diego: Academic Press.

[CIT0009] Chen X-Y, Kim J-Y 2009 Callose synthesis in higher plants. Plant Signaling and Behavior 4, 489–492. 19816126 10.4161/psb.4.6.8359 PMC2688293

[CIT0010] Clark NM, Hinde E, Winter CM, Fisher AP, Crosti G, Blilou I, Gratton E, Benfey PN, Sozzani R 2016 Tracking transcription factor mobility and interaction in Arabidopsis roots with fluorescence correlation spectroscopy. eLife 5, e14770. 27288545 10.7554/eLife.14770 PMC4946880

[CIT0011] Cole LJ, McCracken D, Foster GN, Aitken MN 2001 Using Collembola to assess the risks of applying metal-rich sewage sludge to agricultural land in western Scotland. Agriculture, Ecosystems and Environment 83, 177–189.

[CIT0012] Colzi I, Pignattelli S, Giorni E, Papini A, Gonnelli C 2015 Linking root traits to copper exclusion mechanisms in *Silene paradoxa* L. (Caryophyllaceae). Plant and Soil 390, 1–15.

[CIT0013] Corbesier L, Vincent C, Jang S, Fornara F, Fan Q, Searle I, Giakountis A, Farrona S, Gissot L, Turnbull C 2007 FT protein movement contributes to long-distance signaling in floral induction of Arabidopsis. Science 316, 1030–1033. 17446353 10.1126/science.1141752

[CIT0014] Cui W, Lee J-Y 2016 Arabidopsis callose synthases CalS1/8 regulate plasmodesmal permeability during stress. Nature Plants 2, 16034. 27243643 10.1038/nplants.2016.34

[CIT0015] Cui W, Wang X, Lee J-Y 2015 Drop-ANd-See: a simple, real-time, and noninvasive technique for assaying plasmodesmal permeability. Methods in Molecular Biology 1217, 149–156. 25287202 10.1007/978-1-4939-1523-1_10

[CIT0016] Drew MC 1975 Comparison of the effects of a localized supply of phosphate, nitrate, ammonium and potassium on the growth of the seminal root system, and the shoot, in barley. New Phytologist 75, 479–490.

[CIT0017] Doxey AC, Yaish MWF, Moffatt BA, Griffith M, McConkey BJ 2007 Functional divergence in the Arabidopsis β-1,3-glucanase gene family inferred by phylogenetic reconstruction of expression states. Molecular Biology and Evolution 24, 1045–1055. 17272678 10.1093/molbev/msm024

[CIT0018] Enns LC, Kanaoka MM, Torii KU, Comai L, Okada K, Cleland RE 2005 Two callose synthases, GSL1 and GSL5, play an essential and redundant role in plant and pollen development and in fertility. Plant Molecular Biology 58, 333–349. 16021399 10.1007/s11103-005-4526-7

[CIT0019] Gaudioso-Pedraza R, Benitez-Alfonso Y 2014 A phylogenetic approach to study the origin and evolution of plasmodesmata-localized glycosyl hydrolases family 17. Frontiers in Plant Science 5, 212. 24904609 10.3389/fpls.2014.00212 PMC4033164

[CIT0020] Gisel A, Hempel FD, Barella S, Zambryski P 2002 Leaf-to-shoot apex movement of symplastic tracer is restricted coincident with flowering in Arabidopsis. Proceedings of the National Academy of Sciences, USA 99, 1713–1717. 10.1073/pnas.251675698 PMC122256 11818578

[CIT0021] Grotz N, Fox T, Connolly E, Park W, Guerinot ML, Eide D 1998 Identification of a family of zinc transporter genes from Arabidopsis that respond to zinc deficiency. Proceedings of the National Academy of Sciences, USA 95, 7220–7224. 10.1073/pnas.95.12.7220 PMC22785 9618566

[CIT0022] Gruber BD, Giehl RFH, Friedel S, von Wirén N 2013 Plasticity of the Arabidopsis root system under nutrient deficiencies. Plant Physiology 163, 161–179. 23852440 10.1104/pp.113.218453 PMC3762638

[CIT0023] Gutiérrez-Alanís D, Yong-Villalobos L, Jiménez-Sandoval P, Alatorre-Cobos F, Oropeza-Aburto A, Mora-Macías J, Sánchez-Rodríguez F, Cruz-Ramírez A, Herrera-Estrella L 2017 Phosphate starvation-dependent iron mobilization induces CLE14 expression to trigger root meristem differentiation through CLV2/PEPR2 signaling. Developmental Cell 41, 555–570. 28586647 10.1016/j.devcel.2017.05.009

[CIT0024] Holdaway-Clarke TL, Walker NA, Hepler PK, Overall RL 2000 Physiological elevations in cytoplasmic free calcium by cold or ion injection result in transient closure of higher plant plasmodesmata. Planta 210, 329–335. 10664140 10.1007/PL00008141

[CIT0025] Holmgren G, Meyer M, Chaney R, Daniels R 1993 Cadmium, lead, zinc, copper, and nickel in agricultural soils of the United States of America. Journal of Environmental Quality 22, 335–348.

[CIT0026] Horiunova L, Krasylenko YA, Yemets AI, Blume YB 2016 Involvement of plant cytoskeleton in cellular mechanisms of metals toxicity. Cytology and Genetics 50, 57–67. 27266186

[CIT0027] Huang L, Chen X-Y, Rim Y, Han X, Cho WK, Kim S-W, Kim J-Y 2009 Arabidopsis glucan synthase-like 10 functions in male gametogenesis. Journal of Plant Physiology 166, 344–352. 18760496 10.1016/j.jplph.2008.06.010

[CIT0028] Imlau A, Truernit E, Sauer N 1999 Cell-to-cell and long-distance trafficking of the green fluorescent protein in the phloem and symplastic unloading of the protein into sink tissues. The Plant Cell 11, 309–322. 10072393 10.1105/tpc.11.3.309 PMC144181

[CIT0029] Jacobs AK, Lipka V, Burton RA, Panstruga R, Strizhov N, Schulze-Lefert P, Fincher GB 2003 An Arabidopsis callose synthase, GSL5, is required for wound and papillary callose formation. The Plant Cell 15, 2503–2513. 14555698 10.1105/tpc.016097 PMC280557

[CIT0030] Kawade K 2014 Proliferative control of leaf cells through inter-cell-layer AN3 signaling. Plant Morphology 26, 59–63.

[CIT0031] Kim I, Hempel FD, Sha K, Pfluger J, Zambryski PC 2002 Identification of a developmental transition in plasmodesmatal function during embryogenesis in *Arabidopsis thaliana*. Development 129, 1261–1272. 11874921 10.1242/dev.129.5.1261

[CIT0032] Koizumi K, Hayashi T, Wu S, Gallagher KL 2012 The SHORT-ROOT protein acts as a mobile, dose-dependent signal in patterning the ground tissue. Proceedings of the National Academy of Sciences, USA 109, 13010–13015. 10.1073/pnas.1205579109 PMC3420204 22826238

[CIT0033] Kumar Sharma R, Madhoolika A, Marshall F 2007 Heavy metal contamination of soil and vegetables in suburban areas of Varanasi, India. Ecotoxicology and Environmental Safety 66, 258–266. 16466660 10.1016/j.ecoenv.2005.11.007

[CIT0034] Kurata T, Ishida T, Kawabata-Awai C, Noguchi M, Hattori S, Sano R, Nagasaka R, Tominaga R, Koshino-Kimura Y, Kato T 2005 Cell-to-cell movement of the CAPRICE protein in Arabidopsis root epidermal cell differentiation. Development 132, 5387–5398. 16291794 10.1242/dev.02139

[CIT0035] Lequeux H, Hermans C, Lutts S, Verbruggen N 2010 Response to copper excess in *Arabidopsis thaliana*: impact on the root system architecture, hormone distribution, lignin accumulation and mineral profile. Plant Physiology and Biochemistry 48, 673–682. 20542443 10.1016/j.plaphy.2010.05.005

[CIT0036] Levy A, Erlanger M, Rosenthal M, Epel BL 2007 A plasmodesmata-associated β-1,3-glucanase in Arabidopsis. The Plant Journal 49, 669–682. 17270015 10.1111/j.1365-313X.2006.02986.x

[CIT0037] Li X, Poon C-S, Liu PS 2001 Heavy metal contamination of urban soils and street dusts in Hong Kong. Applied Geochemistry 16, 1361–1368.

[CIT0038] Lin S-I, Chiou T-J 2008 Long-distance movement and differential targeting of microRNA399s. Plant Signaling and Behavior 3, 730–732. 19704842 10.4161/psb.3.9.6488 PMC2634573

[CIT0039] Linkohr BI, Williamson LC, Fitter AH, Leyser HM 2002 Nitrate and phosphate availability and distribution have different effects on root system architecture of Arabidopsis. The Plant Journal 29, 751–760. 12148533 10.1046/j.1365-313x.2002.01251.x

[CIT0040] Liu D, Jiang W, Wang W, Zhao F, Lu C 1994 Effects of lead on root growth, cell division, and nucleolus of *Allium cepa*. Environmental Pollution 86, 1–4. 15091642 10.1016/0269-7491(94)90002-7

[CIT0041] Liu XX, He XL, Jin CW 2016 Roles of chemical signals in regulation of the adaptive responses to iron deficiency. Plant Signaling and Behavior 11, e1179418. 27110729 10.1080/15592324.2016.1179418 PMC4973782

[CIT0042] Mira H, Martínez-García F, Peñarrubia L 2001 Evidence for the plant-specific intercellular transport of the Arabidopsis copper chaperone CCH. The Plant Journal 25, 521–528. 11309142 10.1046/j.1365-313x.2001.00985.x

[CIT0043] Müller J, Toev T, Heisters M, Teller J, Moore KL, Hause G, Dinesh DC, Bürstenbinder K, Abel S 2015 Iron-dependent callose deposition adjusts root meristem maintenance to phosphate availability. Developmental Cell 33, 216–230. 25898169 10.1016/j.devcel.2015.02.007

[CIT0044] Nagajyoti PC, Lee KD, Sreekanth TVM 2010 Heavy metals, occurrence and toxicity for plants: a review. Environmental Chemistry Letters 8, 199–216.

[CIT0045] Pant BD, Buhtz A, Kehr J, Scheible W-R 2008 MicroRNA399 is a long-distance signal for the regulation of plant phosphate homeostasis. The Plant Journal 53, 731–738. 17988220 10.1111/j.1365-313X.2007.03363.x PMC2268993

[CIT0046] Qin R, Wang C, Chen D, Björn LO, Li S 2015 Copper-induced root growth inhibition of *Allium cepa* var. *agrogarum* L. involves disturbances in cell division and DNA damage. Environmental Toxicology and Chemistry 34, 1045–1055. 25639377 10.1002/etc.2884

[CIT0047] Robards AW, Lucas WJ 1990 Plasmodesmata. Annual Review of Plant Biology 41, 369–419.

[CIT0048] Roberts AG, Oparka KJ 2003 Plasmodesmata and the control of symplastic transport. Plant, Cell and Environment 26, 103–124.

[CIT0049] Ross-Elliott TJ, Jensen KH, Haaning KS, et al 2017 Phloem unloading in Arabidopsis roots is convective and regulated by the phloem-pole pericycle. eLife 6, e24125. 28230527 10.7554/eLife.24125 PMC5365319

[CIT0050] Rossow MJ, Sasaki JM, Digman MA, Gratton E 2010 Raster image correlation spectroscopy in live cells. Nature Protocols 5, 1761–1774. 21030952 10.1038/nprot.2010.122 PMC3089972

[CIT0051] Salt DE, Prince RC, Pickering IJ, Raskin I 1995 Mechanisms of cadmium mobility and accumulation in Indian mustard. Plant Physiology 109, 1427–1433. 12228679 10.1104/pp.109.4.1427 PMC157678

[CIT0052] Samardakiewicz S, Krzesłowska M, Bilski H, Bartosiewicz R, Woźny A 2012 Is callose a barrier for lead ions entering *Lemna minor* L. root cells?. Protoplasma 249, 347–351. 21590317 10.1007/s00709-011-0285-2 PMC3305872

[CIT0053] Sancenón V, Puig S, Mateu-Andrés I, Dorcey E, Thiele DJ, Peñarrubia L 2004 The Arabidopsis copper transporter COPT1 functions in root elongation and pollen development. Journal of Biological Chemistry 279, 15348–15355. 14726516 10.1074/jbc.M313321200

[CIT0054] Schönknecht G, Brown JE, Verchot-Lubicz J 2008 Plasmodesmata transport of GFP alone or fused to potato virus X TGBp1 is diffusion driven. Protoplasma 232, 143–152. 18767215 10.1007/s00709-008-0293-z

[CIT0055] Schulz A 1995 Plasmodesmal widening accompanies the short-term increase in symplasmic phloem unloading in pea root tips under osmotic stress. Protoplasma 188, 22–37.

[CIT0056] Seregin IV, Kozhevnikova AD, Kazyumina EM, Ivanov VB 2003 Nickel toxicity and distribution in maize roots. Russian Journal of Plant Physiology 50, 711–717.

[CIT0057] Shimotohno A, Sotta N, Sato T, De Ruvo M, Marée AFM, Grieneisen VA, Fujiwara T 2015 Mathematical modeling and experimental validation of the spatial distribution of boron in the root of *Arabidopsis thaliana* identify high boron accumulation in the tip and predict a distinct root tip uptake function. Plant and Cell Physiology 56, 620–630. 25670713 10.1093/pcp/pcv016 PMC4387314

[CIT0058] Sivaguru M, Fujiwara T, Šamaj J, Baluška F, Yang Z, Osawa H, Maeda T, Mori T, Volkmann D, Matsumoto H 2000 Aluminum-induced 1→3-β-d-glucan inhibits cell-to-cell trafficking of molecules through plasmodesmata. A new mechanism of aluminum toxicity in plants. Plant Physiology 124, 991–1006. 11080277 10.1104/pp.124.3.991 PMC59199

[CIT0059] Steinhorst L, Kudla J 2013 Calcium and reactive oxygen species rule the waves of signaling. Plant Physiology 163, 471–485. 23898042 10.1104/pp.113.222950 PMC3793029

[CIT0060] Takahashi H, Yamazaki M, Sasakura N, Watanabe A, Leustek T, de Almeida Engler J, Engler G, Van Montagu M, Saito K 1997 Regulation of sulfur assimilation in higher plants: a sulfate transporter induced in sulfate-starved roots plays a central role in *Arabidopsis thaliana*.Proceedings of the National Academy of Sciences, USA 94, 11102–11107. 10.1073/pnas.94.20.11102 PMC23632 9380766

[CIT0061] Thiele K, Wanner G, Kindzierski V, Jurgens G, Mayer U, Pachl F, Assaad FF 2009 The timely deposition of callose is essential for cytokinesis in Arabidopsis. The Plant Journal 58, 13–26. 19067977 10.1111/j.1365-313X.2008.03760.x

[CIT0062] Töller A, Brownfield L, Neu C, Twell D, Schulze-Lefert P 2008 Dual function of Arabidopsis glucan synthase-like genes GSL8 and GSL10 in male gametophyte development and plant growth. The Plant Journal 54, 911–923. 18315544 10.1111/j.1365-313X.2008.03462.x

[CIT0063] Vatén A, Dettmer J, Wu S, et al 2011 Callose biosynthesis regulates symplastic trafficking during root development. Developmental Cell 21, 1144–1155. 22172675 10.1016/j.devcel.2011.10.006

[CIT0064] Vert G, Briat J-F, Curie C 2001 Arabidopsis IRT2 gene encodes a root-periphery iron transporter. The Plant Journal 26, 181–189. 11389759 10.1046/j.1365-313x.2001.01018.x

[CIT0065] Vert GA, Briat J-F, Curie C 2003 Dual regulation of the Arabidopsis high-affinity root iron uptake system by local and long-distance signals. Plant Physiology 132, 796–804. 12805609 10.1104/pp.102.016089 PMC167019

[CIT0066] Wang N, Fisher DB 1994 The use of fluorescent tracers to characterize the post-phloem transport pathway in maternal tissues of developing wheat grains. Plant Physiology 104, 17–27. 12232057 10.1104/pp.104.1.17 PMC159158

[CIT0067] Ward JT, Lahner B, Yakubova E, Salt DE, Raghothama KG 2008 The effect of iron on the primary root elongation of Arabidopsis during phosphate deficiency. Plant Physiology 147, 1181–1191. 18467463 10.1104/pp.108.118562 PMC2442553

[CIT0068] Williamson LC, Ribrioux SPCP, Fitter AH, Ottoline Leyser HM 2001 Phosphate availability regulates root system architecture in Arabidopsis. Plant Physiology 126, 875–882. 11402214 10.1104/pp.126.2.875 PMC111176

[CIT0069] Wolf S, Lucas WJ, Deom CM, Beachy RN 1989 Movement protein of tobacco mosaic virus modifies plasmodesmatal size exclusion limit. Science 246, 377–379. 16552920 10.1126/science.246.4928.377

[CIT0070] Xie B, Wang X, Zhu M, Zhang Z, Hong Z 2011 CalS7 encodes a callose synthase responsible for callose deposition in the phloem. The Plant Journal 65, 1–14. 21175885 10.1111/j.1365-313X.2010.04399.x

[CIT0071] Zambryski P 2004 Cell-to-cell transport of proteins and fluorescent tracers via plasmodesmata during plant development. Journal of Cell Biology 164, 165–168. 14734529 10.1083/jcb.200310048 PMC2172327

[CIT0072] Zavaliev R, Epel BL 2015 Imaging callose at plasmodesmata using aniline blue: quantitative confocal microscopy. Methods in Molecular Biology 1217, 105–119. 25287199 10.1007/978-1-4939-1523-1_7

[CIT0073] Zavaliev R, Levy A, Gera A, Epel BL 2013 Subcellular dynamics and role of Arabidopsis β-1,3-glucanases in cell-to-cell movement of tobamoviruses. Molecular Plant-Microbe Interactions 26, 1016–1030. 23656331 10.1094/MPMI-03-13-0062-R

[CIT0074] Zavaliev R, Ueki S, Epel BL, Citovsky V 2011 Biology of callose (β-1,3-glucan) turnover at plasmodesmata. Protoplasma 248, 117–130. 21116665 10.1007/s00709-010-0247-0 PMC9473521

[CIT0075] Zhang H, Shi WL, You JF, Di Bian M, Mei Qin X, Yu H, Liu Q, Ryan PR, Yang ZM 2015 Transgenic *Arabidopsis thaliana* plants expressing a β-1,3-glucanase from sweet sorghum (*Sorghum bicolor* L.) show reduced callose deposition and increased tolerance to aluminium toxicity. Plant, Cell and Environment 38, 1178–1188. 10.1111/pce.12472 25311645

