# Supplementary material for: Exposure to heavy metal stress triggers changes in plasmodesmatal permeability via deposition and breakdown of callose
Source: J Exp Bot. 2018 Jun 13;69(15):3715–28. doi: 10.1093/jxb/ery171 (PMC6022669; doi:10.1093/jxb/ery171)
Supplement: Supplementary Tables Figures [file ery171_suppl_supplementary_tables_figures.pdf]

Supplemental Tables

Gene	Allele	Line	Reference	Source
CalS1	cals1-1	SALK_142792	Weier et al., 2016	Jung-Youn Lee, University of Delaware
CalS2	cals2-1	SAIL_1276_E05	Weier et al., 2016	Jung-Youn Lee, University of Delaware
CalS3	cals3-5	SALK_068418	Vaten et al., 2011	Jung-Youn Lee, University of Delaware
CalS4	cals4-1	SALK_009569	This study	ABRC
CalS5	cals5-2	SALK_026354	Dong et al., 2005	ABRC
CalS5	cals5-3	CS68974	Nishiwaka et al., 2005	ABRC
CalS5	cals5-5	SALK_072226	Nishiwaka et al., 2005	ABRC
CalS6	cals6-1	FLAG_335D06	Weier et al., 2016	ABRC
CalS7	cals7-1	SALK_048921	Xie et al., 2011	Jung-Youn Lee, University of Delaware
CalS8	cals8-1	SALK_037603	This study	ABRC
CalS11	cals11-1	SAIL_18_G03	Weier et al., 2016	ABRC
CalS12	cals12-1/pmr4-1	CS3858	Nishimura et al., 2003	Jung-Youn Lee, University of Delaware
CalS12	cals12-2/pmr4-5	CS67146	Nishimura et al., 2003	ABRC
BG_PPAP	bg_ppap-1	SALK_019116	Levy et al., 2007	ABRC
BG_PPAP	bg ppap-2	SAIL_115_G04	Levy et al., 2007	ABRC
At4g16260	bg6-1	SALK_031479	This study	ABRC
At4g16260	bg6-2	SAIL_1280_B05	This study	ABRC
At1g30080	bg7	SALK_078006	This study	ABRC
At4g18340	bg8	SALK_053553	This study	ABRC

Supplemental Table 1. Germplasm used in this study. Loss-of-function alleles, and their sources, used as indicated in text.

Genotype	Stress	Movement of CF	Movement of GFP	Callose Levels	Inhibition of 1˚ Root Growth?
**A.** Wild-type	50 µM Cu	Increased	Increased (RICS)	Decreased	Yes
	600 µM Fe	Decreased	Decreased	Increased	Yes
	150 µM Zn	Increased	No effect	No effect	No

B, *calS5*	50 µM Cu	Not measured	Not measured	Not measured	***Yes, but less severe than wildtype***
	600 µM Fe	Decreased	***No effect***	***Increased but qualitatively different than wildtype***	***Yes, but less severe than wildtype***
calS12	50 µM Cu	Not measured	Not measured	Not measured	Same as wildtype
	600 µM Fe	Decreased	***No effect***	***No Effect***	Same as wildtype

C. *bg_ppap*	50 µM Cu	***No effect***	Not measured	***No effect***	***More severe than wildtype***
	600 µM Fe	Not Tested	Decreased	Increased	Same as wildtype
bg6	50 µM Cu	***No effect***	Not measured	***No effect***	***More severe than wildtype***
	600 µM Fe	Not Tested	Decreased	Increased	Same as wildtype

Table 2. Effects of heavy metal stress on wild-type and mutant roots. Panel A summarizes the effects on wildtype roots. Panels B and C summarize the effects of 50 µM Cu and 600 µM Fe on the callose synthase and *β-1,3-glucanase* lines, respectively. The highlighted cells are different than wildtype.

Supplemental Figures and Legends

Supplemental Figure 1

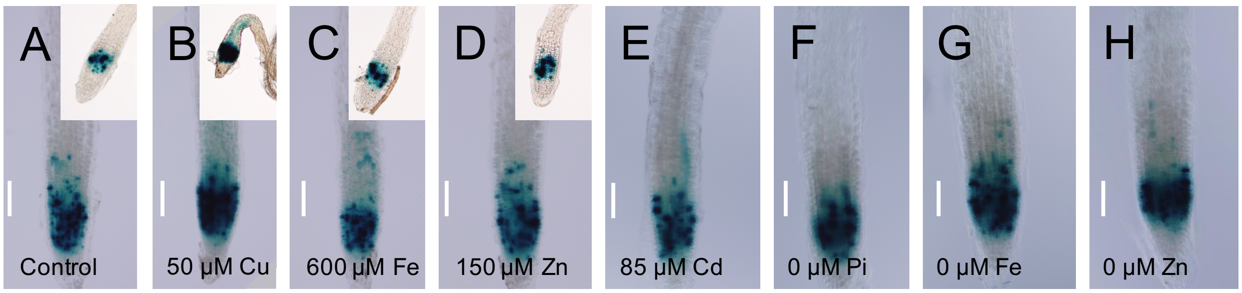

Supplemental Figure 1. Nutrient stress does not inhibit cell divisions. Staining of roots expressing *CycB1;1:GUS* after (A-H) 24 h on (A) control, (B), 50 μM Cu, (C) 600 μM Fe, (D) 150 μM Zn, (E) 85 μM Cd, (F) 0 μM Pi, (G) 0 μM Fe, and (H) 0 μM Zn media. (I-L) GUS staining 3 days after transfer to (I) control conditions, (J), 50 μM Cu, (K) 600 μM Fe, and (L) 150 μM Zn containing media. Scale bar = 60 μm. Inset in (A)-(D) are after 7 days of treatment.

Supplemental Figure 2.

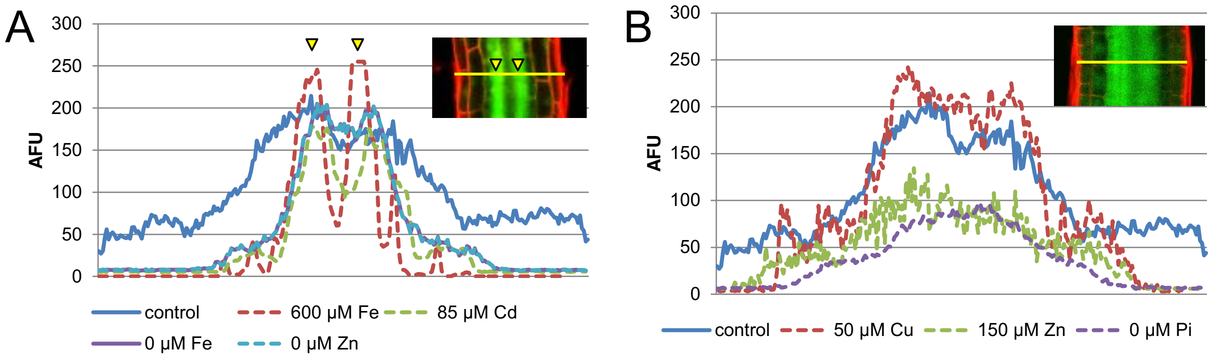

Supplemental Figure 2. Fluorescent profile of GFP as measured radially across the root transition zone. Arbitrary fluorescence units (AFU) of GFP as measure in a 6 day old roots 24 h after growth on media that (A) decreases the QC to stele ratio of GFP fluorescence (600 µM iron, 85 µM cadmium, 0 µM iron or 0 µM zinc; as labeled) or (B) has no effect on movement of GFP out of the phloem (50 µM copper, 150 µM zinc and 0 µM Pi). Control roots are shown in both graphs.

Supplemental Figure 3.

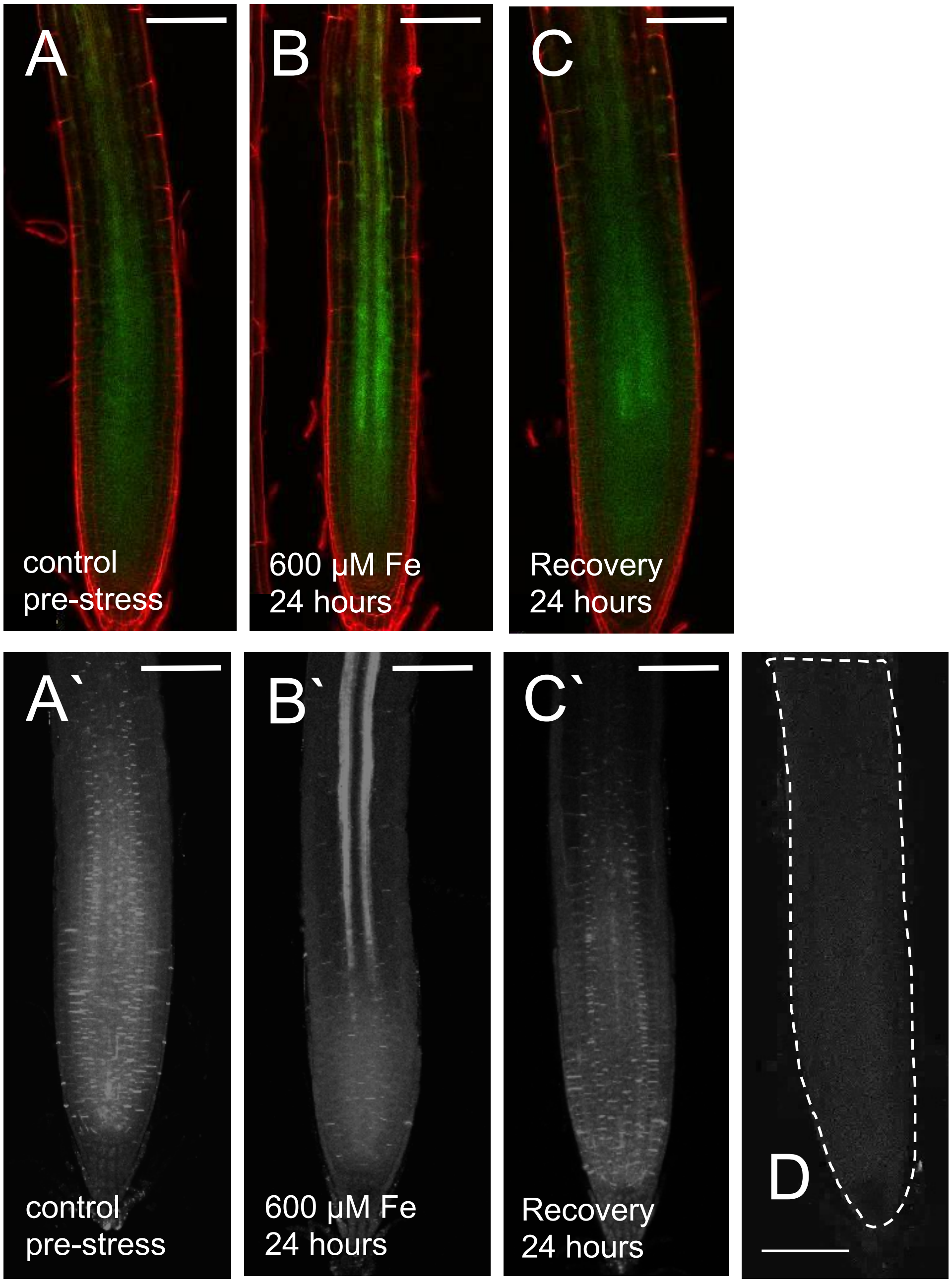

Supplemental Figure 3. The restriction of GFP movement in response to 600 µM iron is reversible and correlates with the presence of callose in the phloem. (A -C) *SUC2:GFP* seedlings stained with (A`-C`) aniline blue. (A and A') prior to iron treatment, (B and B') after 24 hours on 600 µM iron, and (C and C') after 24 h recovery on normal media for 24 hours. (Scale bar 100 µm). (D). Control root (outlined) imaged without aniline blue to show autofluorescence.

Supplemental Figure 4.

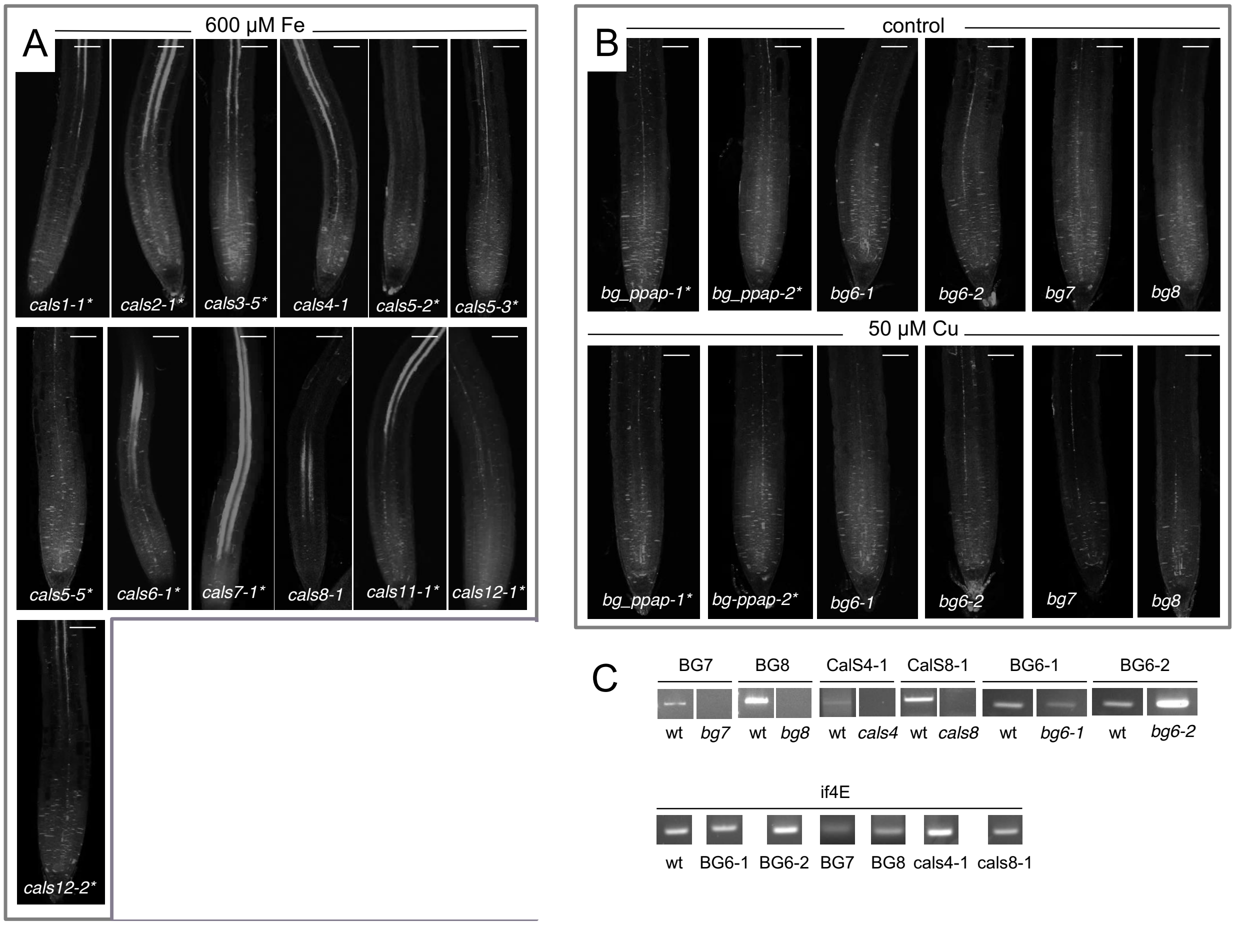

Supplemental Figure 4. Quantification of callose in the *calS* and *β-1,3-glucanase* lines that respond normally to excess iron and copper. Aniline blue staining of roots in the indicated genotypes, 24 h after treatment with (A) 600 µM iron, or (B) 50 µM copper. Scale bar 75um. (C) For each of the *calS* and *β-1,3*-glucanase lines, reverse transcription PCR was used to quantify mRNA levels. Gel images are after 30 cycles. Alleles designated with asterisks indicate already published lines.

Supplemental Figure 5.

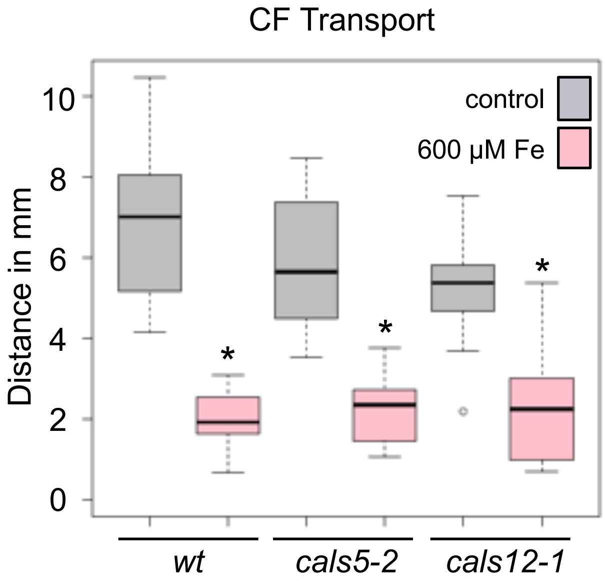

Supplemental Figure 5. Movement of CF is not rescued in the *cals5-2* and *cals12-1* lines. The box plots show the average distance that CF moves shootwards in roots grown for 24 h on 600 µM Fe (Wilcoxan rank sum test, $p<0.05$, n = 25 or greater). Circle outside of box represents outlier.

Supplemental Figure 6.

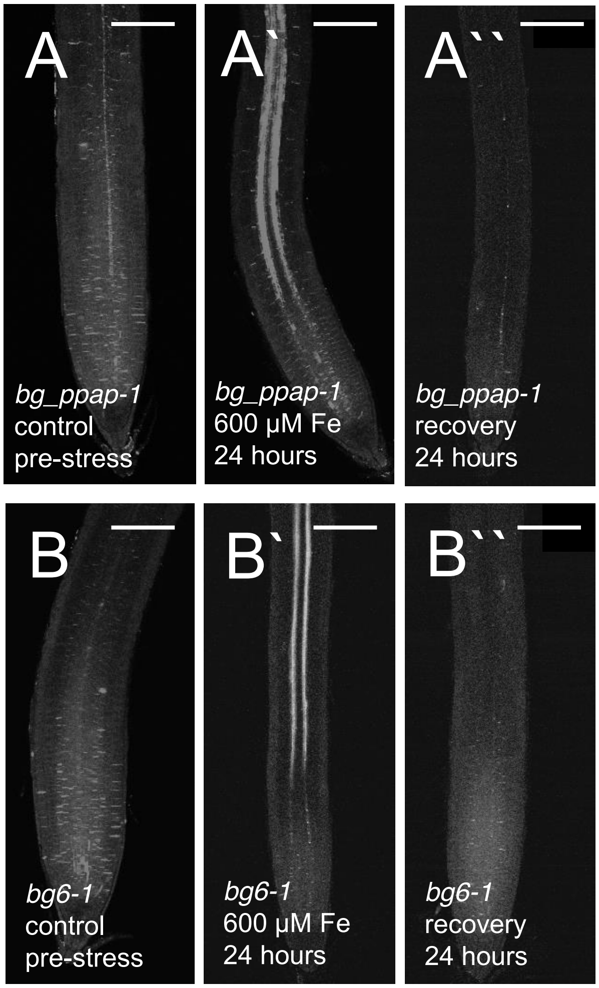

Supplemental Figure 6. The β-1,3-glucanases, *BG_PPAP* and *BG6* are not required for recovery of roots from 600 µM iron. Aniline blue staining of (A-A``) *bg_ppap-1* and (B-B``) *bg6-1* (A and B) prior to stress exposure, (A` and B`) after incubation for 24 h on medium containing 600 µM Fe and (A`` and B``) after recovery for 24 h recovery on control media. Scale bar 100 µm.

Supplemental Figure 7.

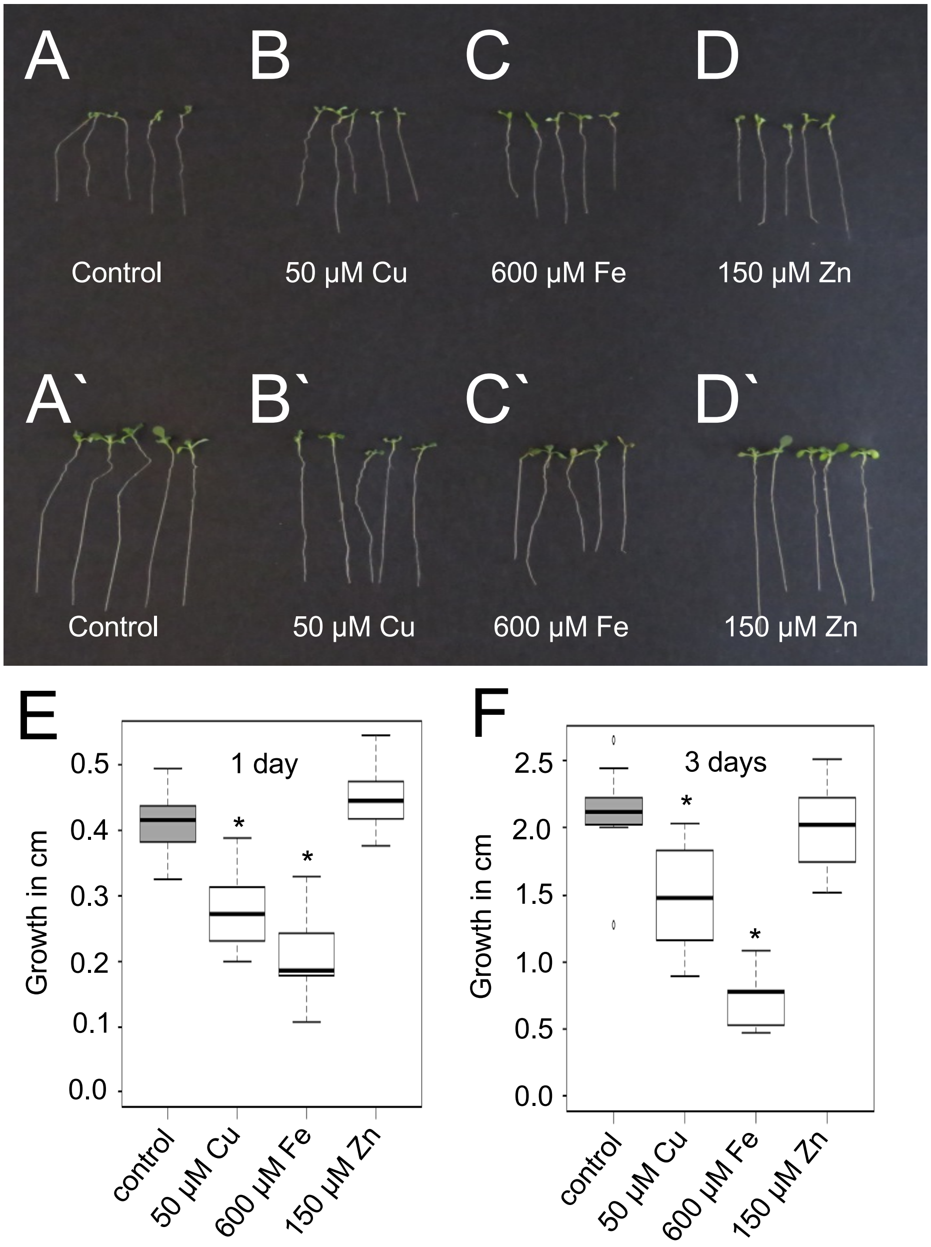

Supplemental Figure 7. Growth of the primary root is inhibited by excess copper or iron. Seedlings grown for (A-D) 1 day or (A`-D`) 3 days on media containing excess copper, iron, or zinc as labeled. (E-F) Increase in the length of the primary root during (E) 1 day of treatment as labeled, or (F) 3 days. Asterisks indicate significant difference from control (2-tailed t-test, $p<0.05$, $n > 14$).

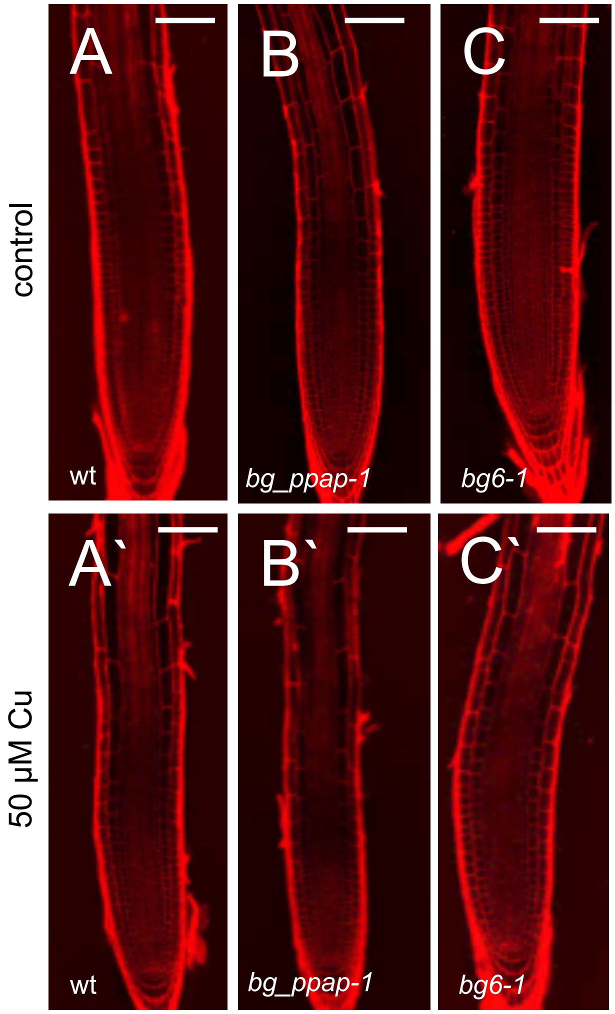

Supplemental Figure 8.

Supplemental Figure 8. Cellular phenotypes and growth of β-1,3-glucanase mutants on 50 µM copper. (A and A`) wildtype, (B and B`) *bg_ppap-1*, and (C-C`) *bg6-1* treated for 24 hours with (A)-(C) control media or (A`)-(C`) 50 µM copper media. Scale bar 100 µm.
